# Transcriptional dynamics and chromatin accessibility in the regulation of shade-responsive genes in Arabidopsis

**DOI:** 10.1186/s13059-025-03901-2

**Published:** 2025-12-10

**Authors:** Sandi Paulišić, Alessandra Boccaccini, René Dreos, Giovanna Ambrosini, Nicolas Guex, Ruben Maximilian Benstein, Markus Schmid, Christian Fankhauser

**Affiliations:** 1 Center for Integrative Genomics, Genopode Building, University of Lausanne, Lausanne, 1015 Switzerland; 2 Bioinformatics Competence Centre, EPFL, Lausanne, Switzerland; 3 Bioinformatics Competence Centre, University of Lausanne, Lausanne, Switzerland; 4 Department of Plant Physiology, Umeå Plant Science Centre, Umeå University, Umeå, 901 87 Sweden; 5 Department of Plant Biology, Linnean Center for Plant Biology, Swedish University of Agricultural Sciences, Uppsala, S-750 07 Sweden; 6 Present Address: Unit of Food Science and Nutrition, Department of Science and Technology for Humans and the Environment, Università Campus Bio-Medico Di Roma, Rome, 00128 Italy; 7 Present Address: HAYA Therapeutics SA, Route De La Corniche 5B, Batiment Alanine, Epalinges, Lausanne, 1066 Switzerland

**Keywords:** Chromatin accessibility, Transcription, Shade, PIF7, Arabidopsis, ATAC-seq

## Abstract

**Background:**

Open chromatin regions host DNA regulatory motifs that are accessible to transcription factors and the transcriptional machinery. In Arabidopsis, responses to light are heavily regulated at the transcriptional level. Shade, for example, can limit photosynthesis and is rapidly perceived by phytochromes as a reduction of red to far-red light ratio (LRFR). Under shade, phytochromes become inactive, enabling PHYTOCHROME INTERACTING FACTORs (PIFs), particularly PIF7, to promote genome-wide reprogramming essential for LRFR responses. An initial strong and fast regulation of shade-responsive genes is followed by attenuation of this response under prolonged shade.

**Results:**

To determine whether the transcriptional response to shade depends on chromatin accessibility, we use ATAC-seq to profile the chromatin of seedlings exposed to short (1 h) and long (25 h) simulated shade. We find that PIF7 binding sites are accessible for most early target genes before LRFR treatment. The transcription pattern of most acute shade-responsive genes correlates with a rapid increase in PIF levels and chromatin association at 1 h, and its decrease at 25 h of shade exposure. For a small subset of acutely responding genes, PIFs also modulate chromatin accessibility at their binding sites early and/or late in the response to shade.

**Conclusions:**

Our results suggest that in seedlings a state of open chromatin conformation allows PIFs to easily access and recognize their binding motifs, rapidly initiating gene expression triggered by shade. This transcriptional response primarily depends on a transient increase in PIF stability and gene occupancy, accompanied by changes in chromatin accessibility in a minority of genes.

**Supplementary Information:**

The online version contains supplementary material available at 10.1186/s13059-025-03901-2.

## Background

In Arabidopsis, a battery of photoreceptors is employed to sense the presence of neighbors by monitoring light quality [1–3]. In dense plant communities, phytochromes perceive the neighbor threat as lower red (R) to far-red (FR) light ratio (LRFR), which signals the risk of being outgrown and shaded by neighbors. The pool of active phyB is then reduced and lifts the repression of PHYTOCHROME INTERACTING FACTORS (PIFs) to promote the shade avoidance response [4, 5]. Active phyB inhibits PIF function by controlling their rapid turnover (e.g., PIF4 and PIF5), and DNA binding and/or transactivation activity [6–13]. However, PIF7 regulation by phyB differs, as it does not involve rapid turnover. This may involve UBIQUITIN-SPECIFIC PROTEASE12 (UBP12) and UBP13 which promote de-ubiquitination of PIF7 [14].

Transcriptional responses to neighbor threat (LRFR) are tightly regulated by PIFs, with a major role established for PIF7 [15, 16]. Exposure to LRFR triggers accumulation and binding of PIFs to G-box and PBE-box motifs initiating rapid expression reprogramming. This affects numerous genes essential for organ elongation and leaf repositioning [15, 17–25]. Among them, auxin biosynthesis YUCCA genes are rapidly induced and raise auxin levels [15, 24, 26] in the cotyledons of seedlings. Auxin is subsequently transported to the hypocotyl, where it promotes cell elongation [27, 28]. Transcriptomic changes induced by LRFR are very fast, reaching a peak between 15–90 min [22, 29] and are followed by relatively fast physiological adaptations [30–33]. Shade induced transcriptional responses and how they control organ growth and repositioning are quite well understood in Arabidopsis [1, 2, 22, 29, 34–36]. However, the epigenetic mechanisms and chromatin landscape underlying transcriptional regulation remain to be fully investigated.

The roles of H3K4me3 and H3K9ac in gene regulation have often been associated with actively transcribing genes. Among recent studies, Calderon et al. have found that the H3K4me3 levels correlate with the active transcription of PIF-regulated and shade-induced genes [37], consistent with previous research, where high transcriptional activity of a particular gene locus was shown to induce the accumulation of H3K4me3 [38, 39]. Shade also induces a significant increase in H3K9ac levels depending on PIF4, PIF5 and/or PIF7 (absent in the triple *pif4pif5pif7* or *pif457* mutant) [40]. H3K9 hyperacetylation was observed not only on the gene bodies of the PIF7 targets, but also in the regulatory regions of specific genes such as *ATHB2* [40–42]. Interestingly, H3K9 hyperacetylation at PIF7 regulatory regions and target genes precedes H3K4me3 accumulation [37, 40].

Besides histone modifications, the chromatin landscape is heavily modified by ATP-dependent SWI/SNF-type chromatin remodeling complexes [43]. These complexes adjust the position and occupancy of nucleosomes through sliding, eviction, or nucleosome deposition, thereby controlling the accessibility of DNA regulatory regions and, ultimately, gene transcription [44]. For instance, the SWI/SNF-type complex characterized by INO80 replaces the histone variant H2A.Z with the canonical H2A, in contrast to SWR1-containing complexes, which mediate H2A.Z deposition [45–48]. Under shade, PIFs recruit the INO80 complex to shade responsive genes and promote H2A.Z eviction [40]. The interaction is usually mediated by EIN6 ENHANCER (EEN), but EEN-independent mechanisms for H2A.Z removal also exist. INO80 was also found to repress light-induced genes, including *HY5* [49]. Moreover, PIF7 also recruits histone chaperone ANTI‐SILENCING FACTOR 1 (ASF1) and HISTONE REGULATOR HOMOLOG A (HIRA) under shade, and establishes a PIF7‐ASF1‐HIRA regulatory module, involved in increasing the H3.3 levels on a subset of actively transcribed shade‐induced genes [16]. Collectively this suggests that PIFs and particularly PIF7 control transcriptional output in response to shade not only by direct activation or repression of gene transcription, but also through remodeling of the chromatin landscape of its target genes.

This led us to hypothesize that the local chromatin environment of PIF binding sites might also have a role in controlling shade triggered genome reprogramming. In this study we have combined RNA-seq and Assay for Transposase Accessible Chromatin using sequencing (ATAC-seq) to determine if the PIF-regulated transcriptional response to LRFR depends on chromatin accessibility. Using ATAC-seq, transcription data, and ChIP-seq data, we observe changes in chromatin accessibility of a set of PIF-regulated genes. Our data indicate that PIF occupancy changes play a more significant role in driving gene expression changes than does remodeling of chromatin accessibility at PIF-binding sites.

## Results

### PIF7 binding sites are accessible in HRFR

**Fig. 1:**
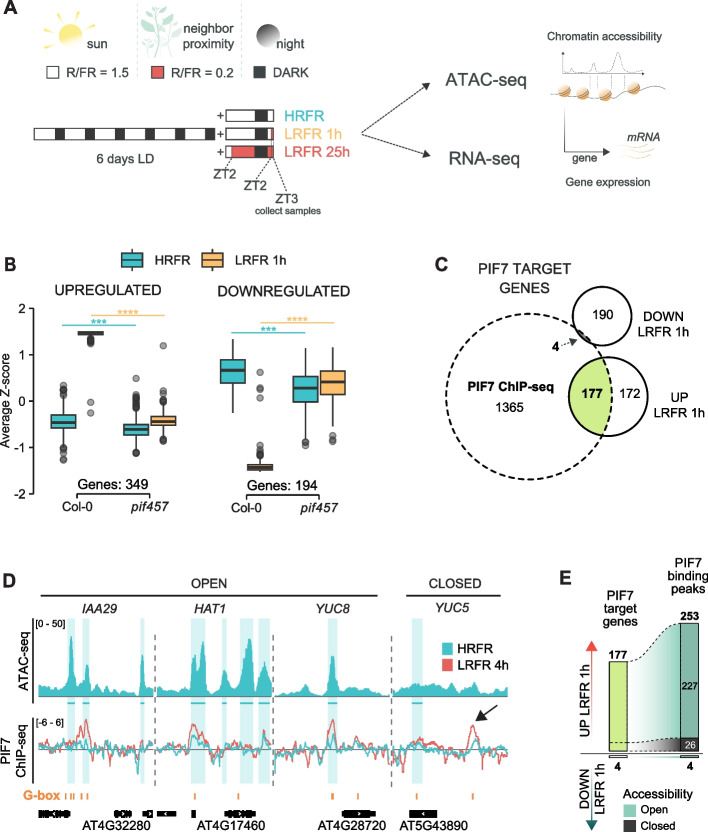
PIF7 binding sites are accessible in HRFR. **A** Experimental set-up for INTACT/ATAC-seq and RNA-seq. Seedlings were grown either in HRFR for 7 days (HRFR), moved to LRFR for 1 h at ZT2 of day 7 (LRFR 1 h) or moved to LRFR at ZT2 of day 6 until day 7 (LRFR 25 h). Samples were collected at ZT3 on day 7 and processed for ATAC-seq or RNA-seq. **B** PIF457 dependent and shade regulated genes upon exposure to 1 h of LRFR from RNA-seq in comparison of Col-0 to *pif457* (padj < 0.05, Log_2_ FC – no cutoff). Z-score represents row-normalized RNA-seq expression (TPM of each gene is subtracted by the row mean and divided by its standard deviation). Asterisks represent statistical significance (ANOVA, post-hoc Tukey HSD, *p < 0.05, **p < 0.01, ***p < 0.001, ****p < 0.0001). **C** PIF7 target genes defined from the overlap of PIF7 binding sites (4 h of LRFR exposure) [40] and PIF457 dependent DEGs at LRFR 1 h (padj < 0.05). Seedlings were grown as in A. **D** IGV browser view of selected PIF7 target genes in HRFR. The upper panel shows averaged ATAC-seq signal from three biological replicates. The lower panel shows PIF7 ChIP-seq signal for HRFR and for 4 h of LRFR exposure normalized to Col-0 IgG using *bamCompare* [40]. Blue shaded areas and lines mark the boundaries of ATAC-seq accessible chromatin regions. The position of G-boxes (CACGTG) is indicated in orange. **E** PIF7 target genes as defined in C are shown with their associated PIF7 binding sites. Accessible (open) PIF7 binding sites are shown in green and non-accessible (closed) sites in gray

To investigate the connection between the transcriptional response to neighbor proximity (using a LRFR light treatment) and chromatin accessibility, we performed ATAC-seq and RNA-seq. We grew the seedlings in LD conditions and exposed them to short (1 h) and long (25 h) LRFR treatments (Fig. 1A) in which neighbor proximity was simulated by supplementing white light with far-red light. We detected 21,303 accessible chromatin sites, ~ 90% of which are present in regions less than 2 kb 5’ of the TSS, hereafter called promoters (Additional file 1: Fig. S1A), suggesting that prior to a LRFR stimulus (in High R/FR abbreviated HRFR), chromatin in gene regulatory regions is generally accessible for the transcriptional machinery and transcription factors. These accessible chromatin regions along the promoters mostly lack DNA methylation as previously described for seedlings grown under similar 16 h-light/8 h-dark cycles (Additional file 1: Fig. S1B) [50].

Since PIF7 is a major transcription factor regulating LRFR responses, with contributions from PIF4 and PIF5 [15, 51] we wanted to specifically assess the chromatin environment of PIF7 binding sites. Therefore, we restricted the analysis to a set of shade-regulated genes bound by PIF7 in LRFR. For this purpose, we reanalyzed a published PIF7 ChIP-seq dataset [40] and compared it to PIF457-regulated genes in response to short term LRFR (Fig. 1B). Although the conditions used by Willige et al., 2021 do not have the same R/FR ratio as our conditions, both yield similarly robust responses [40]. In response to 1 h of LRFR, 349 genes were upregulated, and 194 genes were downregulated in Col-0 compared to *pif457* (Fig. 1B, Additional file 2: Table S2a-b). These acute responding genes comprise most known shade marker genes, including *PIL1*, *ATHB2*, and *HFR1*. We, therefore, defined potential PIF7 targets as genes bound by PIF7 within 3 kb upstream of the TSS or 1 kb downstream of the TES in response to 4 h of LRFR [40] and identified 1546 genes (Fig. 1C). By intersecting these lists, we defined 177 shade-upregulated and 4 -downregulated genes as direct PIF7 targets (Fig. 1C, Additional file 2: Table S3). The majority of PIF7 binding peaks are found on accessible chromatin regions (open) in seedlings grown in HRFR, with only around 10% of them located in non-accessible regions (closed) (Fig. 1D and E). Moreover, by reanalyzing 5, 10 and 30 min ChIP-seq timepoints and comparing them to 4 h of LRFR [40], we noticed that ~ 65–90% of PIF7 target genes are maintained across these timepoints, and that PIF7 binding peaks are predominantly found on accessible chromatin regions (Additional file 1: Fig. S2A-B), reinforcing our idea that PIF7 can easily access its target binding sites prior to a LRFR cue.

### PIFs promote transcriptional response to shade through their increased accumulation and gene occupancy

**Fig. 2:**
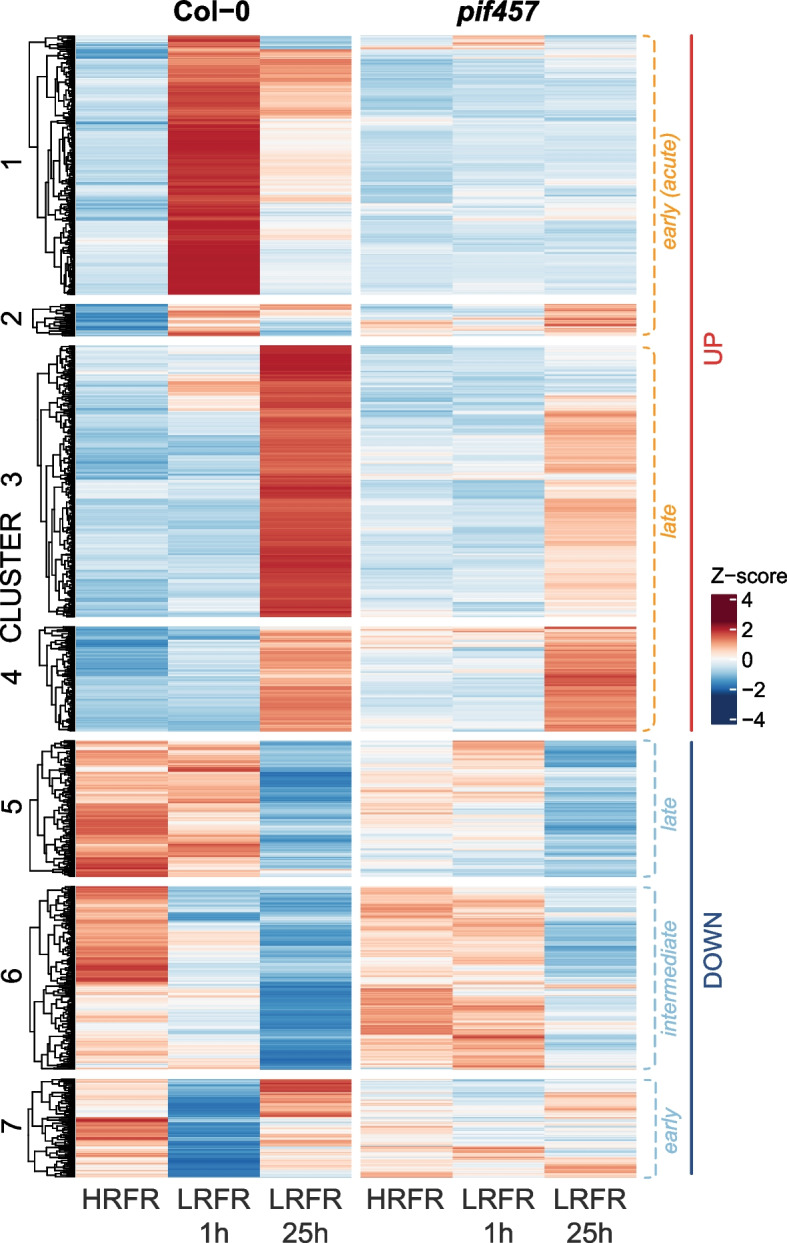
PIFs promote transcriptional response to shade. Heatmap of differentially expressed genes (DEGs) in HRFR, 1 h and 25 h of LRFR in Col-0 and *pif457* mutant (padj < 0.05, abs(Log_2_ FC) > 0.6)

Strong and fast initial regulation of shade responsive genes is followed by an attenuation of this response under prolonged shade [18, 22, 52]. The molecular/regulatory mechanisms underlying this PIF-dependent temporal regulation of transcription are, however, not entirely understood. We, therefore, investigated what may explain this transcription pattern induced by PIFs. Our RNA-seq analysis identified 1148 unique genes that were differentially expressed in Col-0 in response to LRFR exposure for 1 h and/or 25 h (Fig. 2, Additional file 1: Fig. S3A-B, Additional file 2: Table S4a). Clustering analysis of these differentially expressed genes identified 2 clusters (1 and 2) in which the transient upregulation observed in Col-0 was largely abolished in the *pif457* triple mutant (Fig. 2). These 2 clusters are enriched in terms such as shade avoidance and auxin response (Additional file 2: Table S4b), in accordance with the prominent role of these three PIFs in shade responses [51, 53]. A similar transient pattern is observed with downregulated genes of cluster 7 (Fig. 2). In addition, we observed many genes that respond more slowly to LRFR (clusters 3, 4, and 5) and whose expression, with the notable exception of cluster 4, was largely dependent on PIF457. Also, we find significant enrichment related to flavonoid biosynthesis and metabolism in clusters 3 and 4 (Additional file 2: Table S4b).

**Fig. 3:**
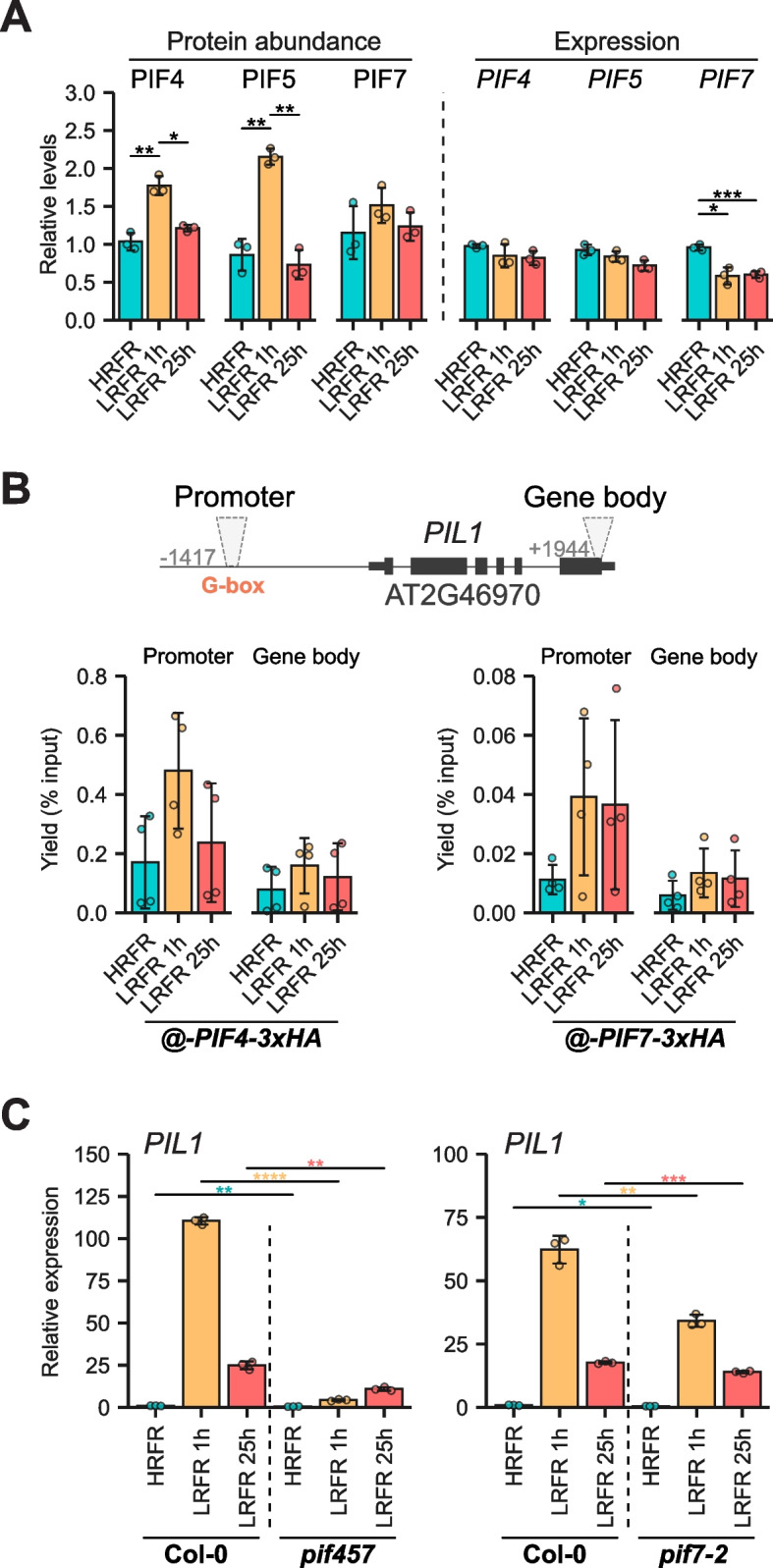
Transient increase in accumulation of PIFs correlates with gene occupancy in response to LRFR. **A** Relative protein abundance of PIF4, PIF5 and PIF7 normalized to DET3 levels (left panel). Relative gene expression of *PIF4*, *PIF5* and *PIF7* normalized to the reference genes *YLS8* and *UBC21* (right panel). Each bar represents the average of three biological replicates, with individual replicates shown as dots. **B** Left panel displays ChIP-qPCR of pPIF4:PIF4-3xHA line (in *pif4-101*) for *PIL1* locus. Right panel displays ChIP-qPCR of pPIF7:PIF7-3xHA line (in *pif7-2*) for *PIL1* locus. Data are from 4 biological replicate and each biological replicate was calculated as the average of minimum two technical qPCR replicates. Bars represent the average of biological replicates. **C** Relative gene expression of *PIL1* in *pif457* (left panel) and *pif7-2* (right panel) mutants. Seedlings were grown either in HRFR for 7 days (HRFR), moved to LRFR for 1 h at ZT2 of day 7 (LRFR 1 h) or moved to LRFR at ZT2 of day 6 until day 7 (LRFR 25 h). Samples were collected at ZT3 on day 7. For gene expression measurements, each bar represents the average of three biological replicates (individual replicates shown as dots) obtained by averaging three technical qPCR replicates. Error bars indicate standard deviation (SD) and asterisks represent statistical significance (Students T-test, *p < 0.05, **p < 0.01, ***p < 0.001, ****p < 0.0001) throughout unless otherwise indicated

Since active phyB regulates the turnover of PIFs and reduces their activity [6–13, 15, 40], we checked the expression and protein levels of PIF4, PIF5, and PIF7 to determine whether PIF levels may underlie the pattern of PIF target gene expression. These PIFs are known to be under strong circadian clock regulation, with a peak in expression around ZT3 and highest protein accumulation at midday in LD conditions [54, 55]. In our conditions, the expression of *PIF4* and *PIF5* was shade-unresponsive, while *PIF7* expression was reduced in response to 1 h and 25 h of LRFR (Fig. 3A), possibly as a compensatory mechanism of the shade avoidance response. Unlike gene expression, PIF4 and PIF5 protein levels significantly increased transiently in response to shade (Fig. 3A), followed by a return to HRFR levels after prolonged LRFR exposure (Fig. 3A). PIF7 total protein levels were overall more stable, accompanied by the disappearance of upper phosphorylated band and a mild and non-significant increase of its levels at 1 h of LRFR (Fig. 3A, Additional file 1: Fig. S3C). A somewhat distinct LRFR regulation of PIF7 activity from the one of PIF4 and PIF5 has been suggested, involving regulation of its phosphorylation status and phyB sequestration into condensates [11, 13, 15, 16, 40]. Next, we examined recruitment of PIFs to their binding sites using ChIP-qPCR. As an example of an early PIF target gene we looked at *PIL1*. We tested PIF binding to canonical CACGTG motifs (G-box) upstream of the *PIL1* TSS to determine whether there was a correlation between PIF protein levels and recruitment to chromatin in response to shade. We observed a correlation between PIF4 protein levels (Fig. 3A) and *PIL1* promoter occupancy (Fig. 3B). Moreover, PIF7 dephosphorylation, and to a less extent its protein levels, followed the increase in *PIL1* promoter occupancy (Fig. 3A-B, Additional file 1: Fig. S4C). The same trend was observed for other PIF-regulated genes (Additional file 1: Fig. S4A-B), indicating that a transient increase in PIF4 protein levels and/or overall change in the phosphorylation status of PIF7 led to higher occupancy of their binding sites, providing a possible explanation for the transient upregulation of early PIF target genes (Fig. 3C).

### Low R/RF promotes dynamic changes of accessible chromatin regions

**Fig. 4:**
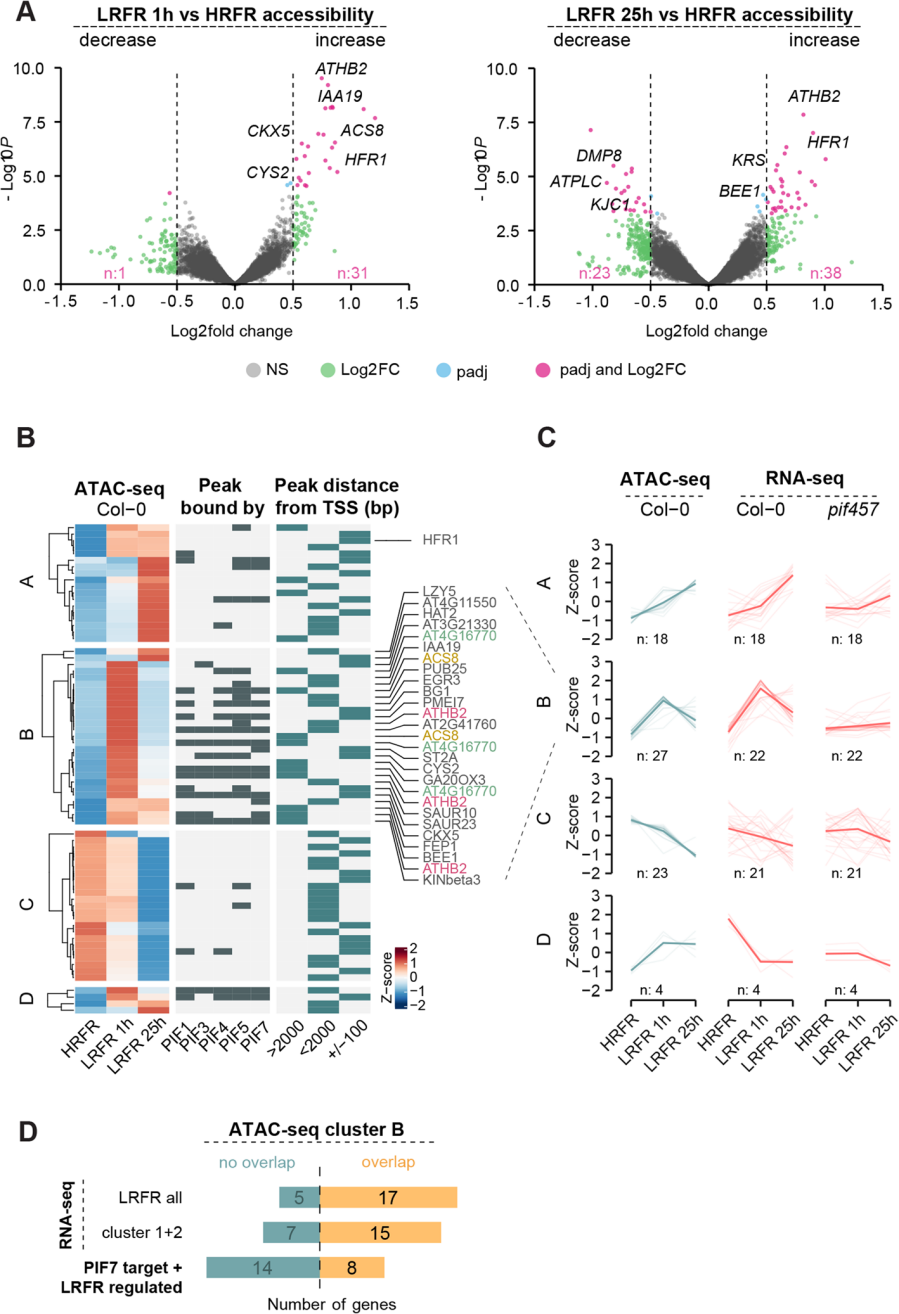
Chromatin accessibility in response to LRFR affects a moderate number of genes. **A** Volcano plots of differentially accessible regions (DARs) in comparisons of 1 h of LRFR (left panel) and 25 h of LRFR (right panel) versus HRFR in Col-0. Statistical cutoff is set at padj < 0.05 and abs(Log_2_ FC) > 0.5. **B** Heatmap of differentially accessible regions (DARs) in Col-0 in HRFR, LRFR 1 h and LRFR 25 h, hierarchically clustered into four distinct clusters based on ATAC-seq and RNA-seq. The middle panel shows DARs bound by PIF1, PIF3, PIF4, and PIF5 [56] and PIF7 [40]. The right panel shows the distance of each DAR to the nearest transcription start site (TSS, defined as ± 100 bp from the start codon). Z-score represents row-normalized ATAC-seq chromatin accessibility (counts per million of each chromatin region are scaled by subtracting the row mean and divided by the row standard deviation). **C** ATAC-seq counts of DARs in Col-0 and the expression of genes in Col-0 and *pif457* associated with the DARs are represented as an average z-score. Z-score represents row-normalized ATAC-seq chromatin accessibility and row-normalized RNA-seq expression (TPM of each gene is subtracted by the row mean and divided by its standard deviation). Thick line is the average trend line. The number of DARs and associated genes in each cluster is displayed below the plots. **D** Number of genes from ATAC-seq cluster B that overlap with all DEGs from RNA-seq or only with acute shade responding clusters 1 and 2 and with PIF7 and shade regulated target genes

To examine the effect of LRFR on accessible chromatin regions we performed differential accessibility analysis of ATAC-seq. In total, we detected 84 differentially accessible regions (DARs), 32 in response to 1 h of LRFR (Fig. 4A, Additional file 1: Fig. S5A, Additional file 2: Table S5a), and 61 in response to 25 h of LRFR (Fig. 4A, Additional file 1: Fig. S5A, Additional file 2: Table S5a). Under short LRFR exposure, the regions were prevalently becoming more accessible compared to HRFR, with less accessible regions appearing after long LRFR exposure (Fig. 4A, Additional file 1: Fig. S5A). Genes associated with DARs that were not expressed in Col-0 were excluded from further analysis, leaving 72 DARs associated with 65 genes (Additional file 2: Table S5b). In general, these DARs were mostly found within 0.1–2 kb 5’ of the TSS [33] and within ± 0.1 kb of the TSS [25], with 17 DARs directly bound by PIF7 (Fig. 4B, Additional file 2: Table S5b).

We performed hierarchical clustering of the 72 DARs and divided them into four distinct clusters, considering chromatin accessibility and gene expression of the associated genes in Col-0 (Fig. 4B). Cluster A consists of DARs that increased accessibility after 25 h of LRFR exposure, with only a few of these sites bound by PIF4 or PIF7 (Fig. 4B), as defined based on the data from [40, 56]. In contrast, cluster B contains DARs that transiently increased accessibility after 1 h of LRFR, and many of these are bound by PIF4 and PIF7 (Fig. 4B). Importantly, the chromatin accessibility pattern of cluster B reflected the transcriptional response of Col-0 and was dependent on PIF457 (Fig. 4C). Most genes in cluster B were regulated by LRFR (Fig. 4D) and included direct PIF7 targets, such as *ATHB2*, *ACS8*, *IAA19*, and *HAT2* (Additional file 1: Fig. S5C). Gene Ontology (GO) enrichment analysis of this cluster confirmed a significant enrichment in terms related to the auxin response and shade avoidance (Additional file 1: Fig. S5D). While transcriptional and accessibility patterns closely matched in clusters A and B, the same cannot be said for cluster C, which were more variable or cluster D, where accessibility increased yet transcription decreased in response to LRFR (Fig. 4C). We note that while for some shade-induced genes, we saw a chromatin accessibility pattern that correlated with gene expression, this group represents less than 5% of transiently shade-induced genes from RNA-seq clusters 1 and 2 (Additional file 2: Table S4a).

Since auxin was found to control chromatin accessibility during developmental reprogramming [57–59], we hypothesized that chromatin accessibility might also be affected by the shade-induced increase in auxin levels. Because SAV3 mediates a critical auxin biosynthetic step in the formation of indole-3-pyruvic acid (IPA) [25], we examined the *sav3-2* mutant by ATAC-seq and compared it to Col-0. The most prominent shade responsive genes such as *ATHB2* and *HFR1*, which are SAV3 independent [15] did not significantly differ between *sav3-2* and Col-0 (Additional file 2: Table S5a-b). However, we observed a weaker response to short term shade for genes present in the PIF dependent cluster B (Additional file 1: Fig. S6A-B). PIF independent clusters showed similar patterns of chromatin accessibility changes in Col-0 and *sav3-2* (Additional file 1: Fig. S6A-B). Yet, the reduced response of cluster B in *sav3-2* suggests a partial dependence on auxin. However, as observed in Col-0, this pattern of chromatin accessibility was only seen for a subset of shade-regulated genes.

In addition to transcriptional changes LRFR promotes various epigenetic changes. Some are well described and induced by PIFs, such as the two marks of active transcription H3K9ac and H3K9me3 [37, 40]. We used published ChIP-seq datasets of these two histone modifications (GSE139296, PRJNA839161) to examine our ATAC-seq gene clusters. We found that, in contrast to other clusters, genes in PIF-dependent cluster B are also more enriched in H3K9ac and H3K9me3 under LRFR (Additional file 1: Fig. S7A-B).

### phyB and PIFs contribute to changes in chromatin accessibility

**Fig. 5:**
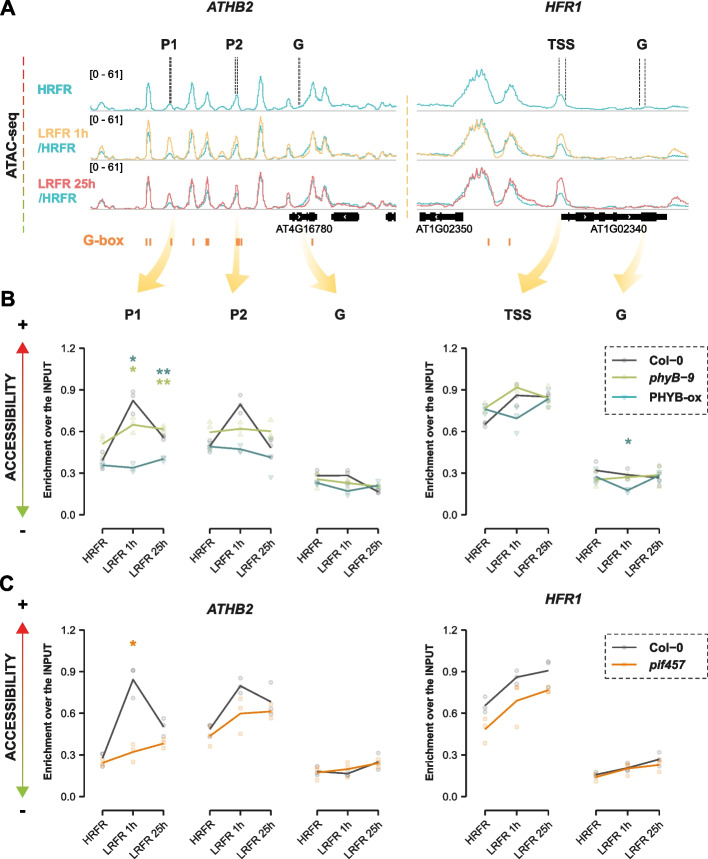
Increase in chromatin accessibility of a set of shade regulated genes is induced by PIFs in response to LRFR. **A** IGV browser view of shade regulated genes *ATHB2* and *HFR1* with changes in chromatin accessibility in response to LRFR in Col-0. Regions tested by CoP-qPCR are marked above the panel. G-boxes are indicated in orange below the panels. ATAC-seq tracks are an average of three biological replicates. **B** Chromatin accessibility of *ATHB2* and *HFR1* assayed by CoP-qPCR in Col-0, *phyB-9* and PHYB-ox (35S:PHYB-GFP). Asterisks represent statistical significance in comparison to Col-0 at the same time point (Student T-test, *p < 0.05, **p < 0.01, ***p < 0.001, ****p < 0.0001). Three biological replicates with the average trend line are presented. **C** Chromatin accessibility of *ATHB2* and *HFR1* assayed by CoP-qPCR in Col-0 and *pif457*. Asterisks represent statistical significance in comparison to Col-0 at the same time point (Student T-test, *p < 0.05, **p < 0.01, ***p < 0.001, ****p < 0.0001). Three biological replicates with the average trend line are presented

To investigate whether the phyB-PIF module is involved in regulating chromatin accessibility in response to LRFR we analyzed DARs of two typical shade-upregulated genes *ATHB2* (from cluster B) and *HFR1* (from cluster A) (Fig. 5A, Additional file 1: Fig. S8C-D). We chose these genes because they have different expression patterns (*ATHB2* expression is more transiently induced by LRFR, while *HFR1* expression remains high in LRFR) and because their DARs have different characteristics: *ATHB2*’s DARs are 5’ upstream of its TSS and are PIF7 binding sites, while *HFR1*’s DAR is at the TSS and is not a PIF7 binding site. Exposure to LRFR led to enhanced accessibility of both *ATHB2* DARs and the *HFR1* TSS (Fig. 5A). Of particular note is that the DAR at *HFR1*’s TSS does not comprise a G-box, while both PIF7 binding sites upstream of *ATHB2* (P1, P2) comprise G-boxes. We compared chromatin accessibility at these DARs with regions on the gene body (G) of both genes, which were less accessible and did not change in response to LRFR (Fig. 5A). Using CoP-qPCR, a method to isolate accessible chromatin regions and compare them to the input to test for enrichment [60], we were able to confirm a significant increase in accessibility of *ATHB2* and *HFR1* DARs in Col-0, validating the ATAC-seq results (Fig. 5B). Since these changes were LRFR-mediated, we first decided to test the role of phyB using both over-expressors and a loss-of-function mutant. Overexpression of phyB resulted in a strong repression of hypocotyl elongation in both HRFR and LRFR, while the absence of phyB promoted hypocotyl elongation (Additional file 1: Fig. S8A-B). In addition, expression of the shade-marker genes *PIL1* and *HFR1* was de-repressed in *phyB-9*, while strongly reduced in the over-expression line (Additional file 1: Fig. S8C-D). In *phyB-9* mutant, *ATHB2* DARs were already more accessible in HRFR and remained more accessible in all conditions (Fig. 5B). The same tendency was observed at the *HFR1* TSS in the *phyB-9* mutant (Fig. 5B), although this region is not directly bound by PIFs. In contrast, phyB overexpression prevented both *ATHB2* DARs from increasing their accessibility in response to shade (Fig. 5B).

Considering PIFs as major targets of phyB regulation and recent findings indicating a role of PIFs in altering the chromatin landscape [6, 7, 9, 11, 13, 40, 61–63], we hypothesized that the effect of phyB on chromatin accessibility may be achieved through PIFs. We, therefore, determined the role of PIF457 in LRFR-induced hypocotyl elongation, expression of shade marker genes, and DARs by using the *pif457* triple mutant (Additional file 1: Fig. S8, Fig. 5C). The response of both *ATHB2* DARs to LRFR was strongly reduced in *pif457*, indicating that PIFs are necessary for the increase in their accessibility (Fig. 5C). The same effect was seen for *IAA19* (Additional file 1: Fig. S8E-F). Interestingly, we also observe overall reduced accessibility of *HFR1* TSS in *pif457*, suggesting a strong effect of PIF457 on this region, although the response to LRFR maintained a similar magnitude (Fig. 5C).

Given the known functional interaction between PIF7 and INO80 [40], we decided to use *ino80-7* mutant to test if chromatin accessibility of shade regulated genes is influenced by INO80 complex. In response to LRFR, *ino80-7* showed an impaired hypocotyl elongation as previously shown for other *ino80* allele [40], but the expression of tested shade regulated genes, except for *HFR1*, was comparable to Col-0 (Additional file 1: Fig. S9A-B). We observed that chromatin accessibility of the tested DARs was also similar to Col-0 (Additional file 1: Fig. S9C-D). Overall, the data suggest that INO80 does not define accessibility of PIF7 binding sites.

*HFR1* has a particular transcription pattern that remains at high levels even after 25 h of LRFR (Additional file 1: Fig. S10C-D) [18]. This pattern correlates with the increased accessibility of its TSS. To investigate the regulation of *HFR1* TSS accessibility and a potential connection between chromatin accessibility and transcription, we investigated HY5 as a potential candidate. HY5 binds to the TSS of *HFR1* [64] and contributes to promoting its expression in response to LRFR (Additional file 1: Fig. S10C), HY5 also repressed hypocotyl elongation in HRFR and LRFR (Additional file 1: Fig. S10A). In addition, we investigated the role of HY5 HOMOLOGUE (HYH), which regulates transcriptional reprogramming together with HY5 [65]. However, in our conditions, *hy5hyh* did not significantly differ from *hy5* (Additional file 1: Fig. S10B, D). Finally, we did not observe significant changes in *HFR1* TSS opening in response to long-term LRFR between Col-0 and *hy5* or *hy5hyh* (Additional file 1: Fig. S10E-F), suggesting that HY5 and HYH do not regulate *HFR1* TSS chromatin accessibility.

## Discussion

Our aim was to determine the potential role of chromatin accessibility during LRFR-induced transcriptional reprogramming. We focused on the roles of PIFs and particularly PIF7, which is known to play a central role in LRFR-induced changes of gene expression [15, 40, 66–68]. Our data indicates that chromatin accessibility is not a major barrier for PIF7 binding and activity prior to shade treatment, given that PIF7 binding sites were accessible prior to LRFR treatment for approximatively 90% of the putative PIF7 target genes (Fig. 1D-E, Additional file 1: Fig. S1A-B, Fig. S2A-B). With relatively easy access to its binding sites, PIF-induced transcription can be very rapid (Fig. 2), consistent with previous publications [18, 22, 29, 40, 69]. Transient shade-regulated changes in transcript abundance observed for hundreds of genes are also unlikely to depend on chromatin accessibility. Indeed, for most PIF7-target genes we did not observe changes in chromatin accessibility in response to LRFR (Fig. 4, Additional file 2: Table S2a-b, S3). Similarly, chromatin accessibility does not seem to explain the distinct response between rapidly LRFR-induced (clusters 1, 2, in Fig. 2) and slowly induced genes (cluster 3, in Fig. 2). Chromatin accessibility was also analyzed during de-etiolation [70], a developmental process characterized by altered expression of thousands of genes [71]. Accessibility changes observed on regulatory regions during de-etiolation are found only for a small subset of differentially expressed genes [70, 71], suggesting that genome-wide transcriptional regulation mediated by light may not need extensive remodeling of chromatin accessibility. Nevertheless, we identified a small subset of shade-regulated PIF7 target genes with changes in chromatin accessibility either at PIF binding sites or at the TSS (Fig. 4). These include well known shade marker genes such as *ATHB2*, *HFR1* and *IAA19*. Most of these genes are found within a slightly larger cluster B of rapidly upregulated genes in response to LRFR with chromatin accessibility changes along their regulatory regions (Fig. 4, Additional file 1: Fig. S1, Additional file 2: Table S5b). We found that these genes are not expressed in a specific cell type under HRFR (i.e., the mesophyll as the most abundant cell type) [72], but they are characterized by two marks of active transcription, H3K9ac and H3K4me3 (Additional file 1: Fig. S7), that are prevalent in PIF regulated genes in response to LRFR [37, 40].

In our study LRFR triggers moderate but significant changes in chromatin accessibility (Fig. 4). To establish what regulates these changes, we investigated auxin as the major driving force of physiological adaptations to shade. Though auxin-related genes show strong transcriptional response under shade [22, 25], the reduction in chromatin accessibility of a set of genes in PIF regulated cluster B observed in *sav3-2* is moderate (Additional file 1: Fig. S6). One hypothesis is that the transient auxin increase during early perception of neighbor threat [73] is not capable of inducing profound remodeling of chromatin accessibility as seen during developmental reprogramming [57–59]. However, given that four genes from cluster B with reduced changes in chromatin accessibility in *sav3-2* also show reduced LRFR-induced expression in this mutant [15], this suggests a link between changes in chromatin accessibility and increased expression in this subset of genes.

Chromatin accessibility at most PIF7 binding sites is stable (Fig. 1), but a group of LRFR regulated genes displays rather dynamic changes at their PIF4/PIF7 binding sites (Fig. 4). Consistent with the role of phyB as the critical regulator of PIFs activity [6–13], we found that PIF7 bound DARs showed increased chromatin accessibility in *phyB-9*, in contrast to overexpressing phyB, supporting the role for PIFs in regulating chromatin accessibility in response to shade (Fig. 5). Whether PIFs can directly remodel the chromatin landscape of the genes they regulate was not fully investigated. However, several publications indicate that PIF-mediated transcription is accompanied by chromatin changes of the transcribed regions [40, 61–63]. In favor of PIFs ability to promote nucleosome remodeling are the described lower chromatin accessibility found at the promoters of *BBX21* and *GLK1* genes in the *pifQ* mutant under darkness [74] and the capacity of the PIF4 and PIF7 to directly interact with the INO80 complex to promote H2A.Z eviction [40, 61–63]. Although PIF7 recruits the INO80 complex in response to shade at transcribed genes [40], we did not observe effect of INO80 on PIF7 binding sites in HRFR or LRFR (Additional file 1: Fig. S9). This may be because the role of INO80 occurs later in the process by changing nucleosome composition as transcription of shade regulated genes occurs. Importantly, we observe a similar trend in chromatin accessibility and transcriptional changes, and for cluster B this seems to correlate with the occupancy of PIF binding sites. Although we are not able to concurrently assess whether differences in the chromatin landscape of PIF binding sites might be associated with the observed transcriptional pattern differences, Willige et al. notice increased hyperacetylation around DNA regulatory regions on the promoters of some shade responsive genes such as *ATHB2* and *HAT3* [40]. This hyperacetylation seems to appear faster than on the gene bodies, even though on much smaller scale [40]. To what extent these changes influence transcriptional response to LRFR needs more attention, as some chromatin modifications occur rapidly [40], while others are trailing marks of active PIF-mediated transcription [37].

Based on our study, we suggest that the chromatin accessibility of PIF binding sites is not a limiting factor in the regulation of gene transcription in response to LRFR, as the sites are easily accessible prior to LRFR exposure. Increase in PIF protein levels and gene occupancy seem to be more predictive of this transcriptional response (Figs. 2 and 3A-C). Several other factors probably contribute to the dynamic regulation of shade responses, including regulation of the phosphorylation status of PIF7 (Additional file 1: Fig. S4C) [11, 15, 16, 40]. Moreover, PIFs are tightly regulated by the circadian clock [75, 76] and modulated by interaction with factors such as HFR1, DELLAs and HY5 [51, 67, 68, 77, 78]. Besides, while HY5 contributes to the transcriptional regulation of *HFR1*, it does not appear to affect HFR1 TSS chromatin accessibility (Additional file 1: Fig. S10), suggesting a primary role for PIFs. More genome wide studies focused on specific cell types are needed to assess PIF7 mediated chromatin remodeling, particularly around critical DNA regulatory motifs.

## Conclusions

Our study highlights the regulation of transcriptional responses to shade by increasing PIF occupancy at already accessible chromatin regions, with a moderate remodeling of that chromatin landscape. We propose that shade-mediated transcriptional regulation may not require extensive remodeling of DNA accessibility and is instead confined to a small subset of genes. These genes might share specific genomic features that we are currently unable to identify. While histone modifications of PIF regulated genes are significant indicator of their transcriptional activity, the main regulatory force of PIF-induced gene regulation appears to be PIF protein dynamics and their interaction with the pre-existing accessible chromatin landscape.

## Methods

### Plant material

*Arabidopsis thaliana* pPIF7:PIF7-3xHA (in *pif7-2*), pPIF5:PIF5-3xHA (in *pif5-3*), pPIF4:PIF4-3xHA (in *pif4-101*) and p35S:PHYB-GFP (PHYBox, in *phyB-9*) lines and *pif7-2*, *pif4-101 pif5-3 pif7-1* (*pif457*), *phyB*-*9*, *sav3-2*, *ino80-7*, *hy5* and *hy5hyh* mutants been previously described [25, 52, 54, 79–85].

### Growth conditions and light treatments

The Arabidopsis thaliana seedlings used in this study, except for hypocotyl elongation, were grown on ½ MS plates (without sucrose) for 7 days at 21 °C under 16 h light/8 h dark conditions (high Red/Far-Red light ratio—HRFR) or treated with low Red/Far-Red light (LRFR). Seedlings were treated with LRFR from ZT2 of day 6 until ZT3 of day 7 (LRFR 25 h) or from ZT2 until ZT3 of day 7 (LRFR 1 h). HRFR ratio corresponds to approximately 1.5 and a total fluence rate of PAR ~ 47–50 µmol/m^−2^ s^−1^. The ratio of LRFR treatment was from 0.13–0.2. For hypocotyl elongation experiments, seedlings were grown on ½ MS plates (without sucrose) for 7 days at 21 °C in HRFR or treated with LRFR from day 4 until day 7. Plants for seed production were grown on soil under 16 h light/8 h dark conditions in walk-in growth chambers.

### Generation of transgenic lines for INTACT-ATAC

For the INTACT-ATAC, the lines expressing both the pUBQ10::BirA and pUBQ10::NTF transgenes were generated in WT Col-0 and introduced into the *sav3-2* mutant by crossing. Briefly, pUBQ10::BirA was generated as described in [86]. The pUBQ10:NTF construct was generated, as also described in [86], cloning the WPP domain of the Arabidopsis thaliana RAN GTPASE ACTIVATING PROTEIN 1 (RanGAP1; At3g63130) at the N terminus, followed by the enhanced GFP protein (eGFP) and the biotin ligase recognition peptide downstream of the UBQ10 promoter. After crossing with *sav3-2* mutant, plants bearing both the pUBQ10:BirA and pUBQ10:NTF were selected for the resistance to glufosinate (BASTA) and kanamycin, respectively. Moreover, the expression of the biotinylated NTF proteins was tested running total protein extracted from homozygous lines on 4–15% gel, transferred on nitrocellulose membrane and probed with anti-Streptavidin antibody conjugated with HRP for chemiluminescent visualization.

### Hypocotyl measurements

Hypocotyl elongation measurements were done as described previously [52]. In brief, seedlings were grown on ½ MS vertical plates in a growth incubator in HRFR until day 4 (ZT2) and images were taken. Plates with seedlings were transferred to LRFR or kept in HRFR until taking images on day 7 (ZT2). Hypocotyl length was measured with a MATLAB script developed in the C.F. laboratory.

### Reverse Transcription—quantitative PCR (RT-qPCR)

Total Arabidopsis RNA was extracted using Plant RNeasy kit (Qiagen, Cat. No./ID: 74,904) according to the manufacturer’s instructions. cDNA was synthesized using Superscript II Reverse Transcriptase (Invitrogen, Life Technologies) with random oligonucleotides. Quantitative real-time PCR (qPCR) was done in three biological replicates with three technical replicates on QuantStudio 6 Flex Real-Time PCR System (Applied Biosystems). Gene expression data was normalized against UBC and YSL8 genes. Primers used for the qPCR reactions can be found in Additional file 1: Table 1.

### ChIP-qPCR

For one biological replicate, 15 mg of seeds were sown on ½ MS. Seedlings were harvested, frozen in liquid nitrogen and ground with mortar and pestle. Ground powder was transferred to 10 mL of cold EB1 (60 mM HEPES pH 8, 0.4 M sucrose, 10 mM KCl, 10 mM MgCl_2_, 5 mM EDTA, cOmplete™ Mini EDTA-free Protease Inhibitor Cocktail) supplemented with 1% formaldehyde and incubated for 10 min at RT on a rotating wheel. Crosslinking was stopped by adding freshly prepared 2 M glycine to a final concentration of 0.125 M, incubated for 10 min at RT on a rotating wheel. Extracts were filtered through a layer of Miracloth, centrifuged for 10 min at 4000xg at 4 °C and supernatant was removed. Nuclei pellets were resuspended first in 1 mL of EB2 (0.25 M sucrose, 10 mM Tris–HCl pH 8, 1% Triton, 10 mM MgCl_2_, 5 mM beta-mercaptoethanol, cOmplete™ Mini EDTA-free Protease Inhibitor Cocktail), centrifuged for 10 min at 5000xg at 4 °C and then resuspended in 300 µL EB3 (1.7 M sucrose, 10 mM Tris–HCl pH 8, 0.15% Triton, 2 mM MgCl_2_, 5 mM beta-mercaptoethanol, cOmplete™ Mini EDTA-free Protease Inhibitor Cocktail). After centrifugation for 1 h at 16000xg at 4 °C, nuclei pellets were resuspended in 200 µL of NLB (50 mM Tris–HCl pH 8, 10 mM EDTA, 1% SDS, cOmplete™ Mini EDTA-free Protease Inhibitor Cocktail) and sonicated for 15 min on high power (PIF7) and 20 min on low power (PIF4) (SONICATION Bioruptor Digenode: 30 s ON 30 s OFF, 10 cycles).

The chromatin was immunoprecipitated with mouse monoclonal HA-Tag Antibody (F-7) (Santa Cruz Biotechnology, Cat No./ID: sc-7392) coupled to Dynabeads™ Protein A and Protein G mixture (Invitrogen, Cat No./ID: 10001D and 10003D). Chromatin was eluted in EB (1% SDS, 0.1 M NaHCO3) at 65 °C for 15 min twice. Input and IP were reverse crosslinked overnight at 65 °C with NaCl (0.2 M final) then treated with RNase A (Qiagen, Cat No./ID: 19,101) for 30 min at 37 °C, followed by Proteinase K (Fisher Scientific, Cat No./ID: AM2546) for 30 min at 55 °C. DNA was purified with QIAquick PCR Purification Kit (Qiagen, Cat No./ID: 28,104).

Quantitative real-time PCR (qPCR) was done with two to three biological replicates per experiment with three technical replicates on QuantStudio 6 Flex Real-Time PCR System (Applied Biosystems) and normalized against the input. Primers used for the qPCR reactions can be found in Additional file 1: Table 1.

### Western blot—SDS-Page

For protein extractions, 20–25 seedlings were frozen in liquid nitrogen, ground to powder and resuspended in 2 × Laemmli buffer (0.125 M Tris–HCl pH 6.8, 4% SDS, 20% glycerol, 10% beta-mercaptoethanol, bromophenol blue). Extracts were heated for 5 min at 95 °C, spined down and loaded to 4–15% Mini-PROTEAN® TGX™ Precast Protein Gels (Bio-Rad, Cat No./ID: 4,561,086). After semi-dry transfer, the membranes were probed with rat monoclonal anti-HA antibody coupled to HRP (Roche, Cat No./ID: 12,013,819,001). Rabbit polyclonal anti-DET3 (Schumacher et al. 1999) and mouse monoclonal anti-tubulin (Abiocode, Cat No./ID: M0267-1a) antibodies were used for normalization. Chemiluminescence was generated with Immobilon Western Chemiluminescent HRP Substrate on Fujifilm ImageQuant LAS 4000 mini-CCD camera system (GE Healthcare).

### CoP (column purified isolation of regulatory elements)

Accessible chromatin was extracted using a modified CoP method [60]. For one biological replicate, 6–10 mg of seeds were sown on ½ MS. Seedlings were harvested, frozen in liquid nitrogen and ground with mortar and pestle. Ground powder was transferred to 35 mL of cold EB1 (60 mM HEPES pH 8, 1 M sucrose, 5 mM KCl, 5 mM MgCl_2_, 5 mM EDTA, 0.6% Triton-X100, cOmplete™ Mini EDTA-free Protease Inhibitor Cocktail) and incubated with 1% formaldehyde for 10 min at RT on a rotating wheel. Crosslinking was stopped by adding 2.4 mL of freshly prepared 2 M glycine, incubated for 10 min at RT on a rotating wheel. Extracts were filtered through a layer of Miracloth, centrifuged for 20 min at 4000xg at 4 °C and supernatant was removed. Nuclei pellets were resuspended first in 1 mL of EB2 (0.25 M sucrose, 10 mM Tris–HCl pH 8, 1% Triton, 10 mM MgCl_2_, 5 mM beta-mercaptoethanol, cOmplete™ Mini EDTA-free Protease Inhibitor Cocktail), centrifuged for 10 min at 12000xg at 4 °C and then resuspended in 300 µL EB3 (1.7 M sucrose, 10 mM Tris–HCl pH 8, 0.15% Triton, 2 mM MgCl_2_, 5 mM beta-mercaptoethanol, cOmplete™ Mini EDTA-free Protease Inhibitor Cocktail). After centrifugation for 1 h at 16000xg at 4 °C, nuclei pellets were resuspended in NLB (50 mM Tris–HCl pH 8, 10 mM EDTA, 1% SDS, cOmplete™ Mini EDTA-free Protease Inhibitor Cocktail) and sonicated for 10 min on high power (SONICATION Bioruptor Digenode: 30 s ON 30 s OFF, 10 cycles).

10% of chromatin was spared as input, the rest was purified using silica membrane columns from Wizard® SV Gel and PCR Clean-Up kit (Promega, Cat No./ID: A9282). Input was treated with RNase A (Qiagen, Cat No./ID: 19,101) for 30 min at 37 °C, followed with Proteinase K (Fisher Scientific, Cat No./ID: AM2546) for 30 min at 55 °C, then at 65 °C overnight. DNA was purified with Wizard® SV Gel and PCR Clean-Up kit and used for quantitative real-time PCR (qPCR). Three biological replicates with three technical replicates were checked on QuantStudio 6 Flex Real-Time PCR System (Applied Biosystems) unless stated otherwise. Enrichment over the input was calculated for the chromatin accessibility of each region. Chromatin accessibility was further normalized against two open control regions on the promoters of ACT2 (AT3G18780, region chr3:6,474,579: 6,474,676) and RNA polymerase II transcription elongation factor (AT1G71080 region chr1:26,811,833:26,811,945). Primers used for the qPCR reactions can be found in Additional file 1: Table 1.

### INTACT/ATAC-seq

For each biological replicate of ATAC-seq, 10 mg of seeds were sown on ½ MS plates to obtain around 0.4 g of seedlings. ATAC-seq was performed as described previously [87] with minor modifications. In brief, the tissue was frozen in liquid nitrogen for nuclei isolation using the INTACT method [86]. The frozen tissue was ground with mortar and pestle then transferred to 15 mL tube with NPB (MOPS pH7 20 mM, NaCl 40 mM, KCl 90 mM, EDTA 2 mM, EGTA 0.5 mM, Spermidine 0.5 mM, Spermine 0.2 mM, 0.5 × Complete Protease Inhibitors) and filtered through one layer of Miracloth and 30 µm filter-tubes (Sysmex, Cat No./ID: 04–004–2326). After centrifugation at 1000xg for 10 min at 4 °C, the nuclei were resuspended in 1 mL of NPB and stained with 4,6-diamidino-2-phenylindole, centrifuged at 1000xg for 10 min at 4 °C, then resuspended in 1 mL of NPB.

Then, nuclei were conjugated to Dynabeads™ M-280 Streptavidin (Invitrogen, Cat No./ID: 11205D) and captured on the magnetic rack. Supernatant was removed and beads were washed twice with NPB, then left resuspended in NPB on ice. The purified nuclei (25,000–50,000 nuclei) were incubated with Tagment DNA TDE1 Enzyme and Buffer mix (Illumina, Cat No./ID: 20,034,197) at 37 °C for 30 min. DNA was purified with Wizard SV Gel and PCR clean-up system (Promega, Cat No./ID: A9282). Library was amplified using 2X KAPA HiFi HotStart (Roche, Cat No./ID: KK2601) and Nextera Primer Mix (i7 + i5) for 11 cycles (98 °C for 20’’, 63 °C for 30’’, 72 °C for 30’’; last step 72 °C for 1’). Amplified libraries were purified with ProNEx Size selective Purification System (Promega, Cat No./ID: NG2001) and only libraries with fragments between 100 and 600 bp, peaking at 300 bp were considered. Three biological replicates were used for sequencing on HiSeq 4000 (Illumina).

### RNA-seq

For each biological replicate of RNA-seq, 25–30 seedlings grown on ½ MS plates were harvested, frozen in liquid nitrogen and ground to powder. Total RNAs were extracted using RNeasy Plant Mini Kit (Qiagen, Cat No./ID: 74,904) according to the manufacturer’s instructions. Libraries generated with Illumina Stranded mRNA Prep (Illumina, Cat No./ID: 20,040,534) were sequenced on NovaSeq 6000 (Illumina).

### ATAC-seq analyses

Reads were mapped to the A. thaliana reference genome (TAIR10) using STAR [88]. Duplicates were removed with MarkDuplicates and sorted with SortSam from Picard. Blacklisted regions as defined in [89] were removed with BEDTools intersect [90]. To identify ATAC-seq peaks we employed MACS3 [91] without shifting the reads introduced by Tn5. Consensus peaks were generated based on an overlap of at least 50% of length in all replicates. Only peaks detected in all replicates were considered and chloroplast and mitochondrial peaks were removed. ChIPseeker [92] was used for peak annotation and clusterProfiler package [93] for functional annotation of peaks. Differential accessibility analysis was done with edgeR [94]. Bedgraph files created from BAM files with bamCoverage from deepTools [95] were scaled using –scaleFactor and converted to BigWig format with bedGraphToBigWig.

### RNA-seq analyses

Reads were trimmed with Cutadapt [96], filtered for ribosomal RNA with fastq_screen and further filtered for low complexity with reaper [97]. Reads were aligned to the A. thaliana reference genome (TAIR10) using STAR [88] and counted using htseq-count [98]. Differential gene expression analysis was done using DESeq2 [99] with the threshold of "p.adj < 0.05 and abs(log2FoldChange) > 0.6".

### ChIP-seq re-analysis

PIF7 ChIP-seq data GSE139296 [40] in response to 4 h of LRFR was re-analyzed and annotated with ChIPseeker [92]. We defined PIF7 direct and shade regulated genes by overlapping PIF7 ChIP-seq and RNA-seq. H3K4me3 data from PRJNA839161 project [37] was re-analyzed as follows, reads were aligned to the A. thaliana reference genome (TAIR10) using bowtie2 [100], samtools [101] was used to sort and index BAM files, and MarkDuplicates from Picard Tools to remove duplicates. Averaged bigWig files were used for plotting profiles and heatmaps with plotProfile and plotHeatmap after computeMatrix from deepTools. Published H3K9ac ChIP-seq data GSE139296 [40] was re-analyzed from averaged bigWig files as stated previously. DNA methylation [50] profile plots for Col-0 were plotted from averaged bigWig files (GSE164588) using trackplot [102, 103]. The DNA binding summits of PIF1, PIF3, PIF4 and PIF5 as defined in [56] were used for analyzing the overlap with accessible chromatin regions.

### GO analysis

GO enrichment analysis was performed using compareCluster from the clusterProfiler package [93]. Integrative Genomics Viewer was used to visualize the ATAC-seq and ChIP-seq signals [104].

### Accession numbers for genes mentioned in this article

IAA19 – AT3G15540

YUC8 – AT4G28720

ATHB2 – AT4G16780

PIL1—AT2G46970

PIF4—AT2G43010

PIF5—AT3G59060

PIF7—AT5G61270

HFR1—AT1G02340

HY5—AT5G11260

HYH—AT3G17609

PHYB—AT2G18790

YLS8—AT5G08290

UBC21 (PEX4)—AT5G25760

## Supplementary Information

Additional file 1: Supplementary figures. Fig. S1. Distribution of the accessible sites in the genome. Fig. S2. PIF7 target genes at different times of LRFR exposure. Fig. S3. PIFs promote transcriptional response to shade. Fig. S4. Transient increase in accumulation and stability of PIFs correlates with gene occupancy in response to LRFR. Fig. S5. Chromatin accessibility in response to LRFR affects a moderate number of genes. Fig. S6. Increase in chromatin accessibility in response to LRFR in *sav3-2*. Fig. S7. Marks of active transcription increase in PIF dependent cluster under shade. Fig. S8. Increase in chromatin accessibility of a set of shade regulated genes is induced by PIFs in response to LRFR. Fig. S9. Chromatin accessibility in *ino80-7*mutant. Fig. S10. Increase in chromatin accessibility of HFR1 does not depend on HY5 or HYH [110–113].

Additional file 2: Supplementary tables. Table S1. List of primers used in this study. Table S2a. PIF457 upregulated genes. Table S2b. PIF457 downregulated genes. Table S3. PIF7 direct targets and shade regulated genes. Table S4a. Differentially expressed genes in RNA-seq in response to LRFR. Table S4b. Gene enrichment analysis of the RNA-seq in response to LRFR. Table S4c. TPM of genes from the RNA-seq in response to LRFR. Table S4d. HTSEQ counts of RNA-seq in response to LRFR. Table S5a. Differential analysis of ATAC-seq in response to LRFR. Table S5b. Clustering of differentially accessible regions in ATAC-seq in response to LRFR. Table S5c. MACS3 peak counts for ATAC-seq in response to LRFR

Additional file 3: Review history

## Data Availability

The sequencing data generated in this study is available in the Gene Expression Omnibus database (GEO), under accession no. GSE283129 (110) and accession no. GSE283133 (111). Other datasets used in this study which include ChIP-seq dataset of PIF7, RNA-seq dataset of Col-0 seedlings used for RNA-seq average profile and ChIP-seq dataset of H3K9ac in Col-0 and *pif457* seedlings were obtained from GEO accession no. GSE139296 (40, 112). Methylation dataset of Col-0 seedlings was obtained from GEO accession no. GSE164588 (50, 113). ChIP-seq dataset of H3K4me3 in Col-0 and *pifS* seedlings was obtained from BioProject no. PRJNA839161 (37).
