# Supplementary material for: Transcriptional dynamics and chromatin accessibility in the regulation of shade-responsive genes in Arabidopsis
Source: Genome Biol. 2025 Dec 10;26:422. doi: 10.1186/s13059-025-03901-2 (PMC12690957; doi:10.1186/s13059-025-03901-2)
Supplement: Supplementary file 1 — Additional file 1: Supplementary figures. Fig. S1. Distribution of the accessible sites in the genome. Fig. S2. PIF7 target genes at different times of LRFR exposure. Fig. S3. PIFs promote transcriptional response to shade. Fig. S4. Transient increase in accumulation and stability of PIFs correlates with gene occupancy in response to LRFR. Fig. S5. Chromatin accessibility in response to LRFR affects a moderate number of genes. Fig. S6. Increase in chromatin accessibility in response to LRFR in sav3-2. Fig. S7. Marks of active transcription increase in PIF dependent cluster under shade. Fig. S8. Increase in chromatin accessibility of a set of shade regulated genes is induced by PIFs in response to LRFR. Fig. S9. Chromatin accessibility in ino80-7mutant. Fig. S10. Increase in chromatin accessibility of HFR1 does not depend on HY5 or HYH [110–113]. [file 13059_2025_3901_MOESM1_ESM.pdf]

Additional file 1 (Supplementary figures S1 to S10)

Transcriptional dynamics and chromatin accessibility in the regulation of shade-responsive genes in Arabidopsis

Sandi Paulišić, Alessandra Boccaccini, René Dreos, Giovanna Ambrosini, Nicolas Guex, Ruben Maximilian Benstein, Markus Schmid, Christian Fankhauser

Fig. S1. Distribution of the accessible sites in the genome.

Fig. S2. PIF7 target genes at different times of LRFR exposure.

Fig. S3. PIFs promote transcriptional response to shade.

Fig. S4. Transient increase in accumulation and stability of PIFs correlates with gene occupancy in response to LRFR.

Fig. S5. Chromatin accessibility in response to LRFR affects a moderate number of genes.

Fig. S6. Increase in chromatin accessibility in response to LRFR in *sav3-2*.

Fig. S7. Marks of active transcription increase in PIF dependent cluster under shade.

Fig. S8. Increase in chromatin accessibility of a set of shade regulated genes is induced by PIFs in response to LRFR.

Fig. S9. Chromatin accessibility in *ino80-7* mutant.

Fig. S10. Increase in chromatin accessibility of HFR1 does not depend on HY5 or HYH.

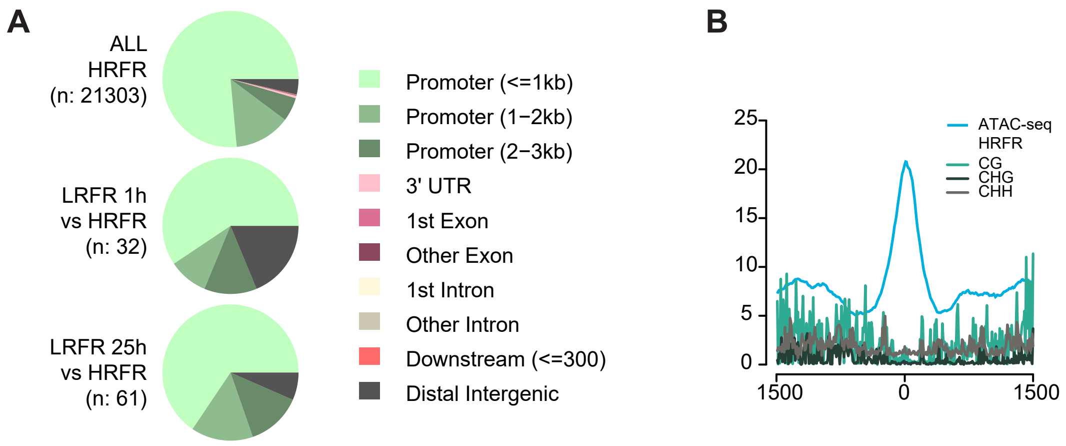

Fig. S1. Distribution of the accessible sites in the genome.

A. Distribution of ATAC-seq peaks across genomic regions.
B. Average ATAC-seq profile plot of 84 DARs in HRFR and average methylation profiles from (Zhou et al., 2022).

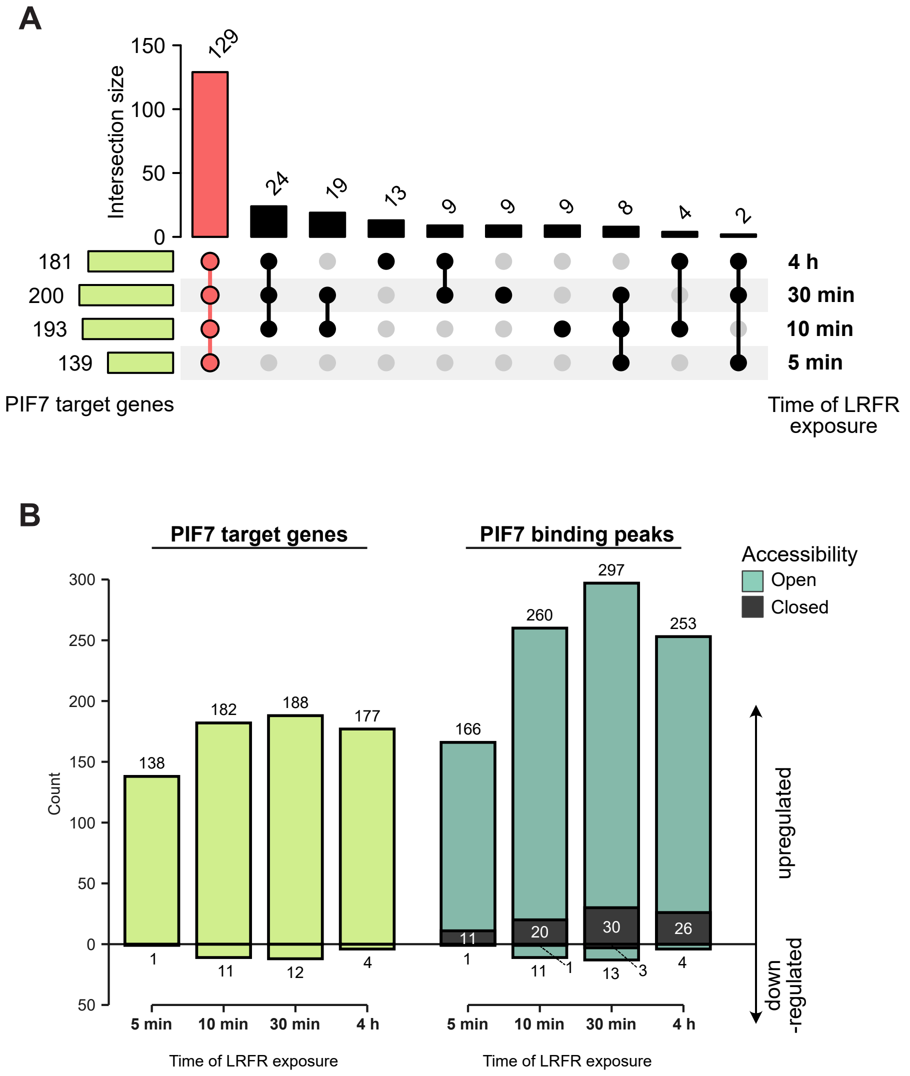

Fig. S2. PIF7 target genes at different times of LRFR exposure.

A. Upset-plot of intersected PIF7 target genes at 5, 10, 30 min and 4 h of LRFR exposure. ChIP-seq data is from Willige et al, 2021.

B. PIF7 target genes (in light green) shown with their associated PIF7 binding sites. Accessible (open) PIF7 binding sites are shown in green and non-accessible (closed) sites in gray. Genes are separated into up- and down-regulated. ChIP-seq data for 5, 10, 30 min and 4 h of LRFR exposure is from Willige et al, 2021.

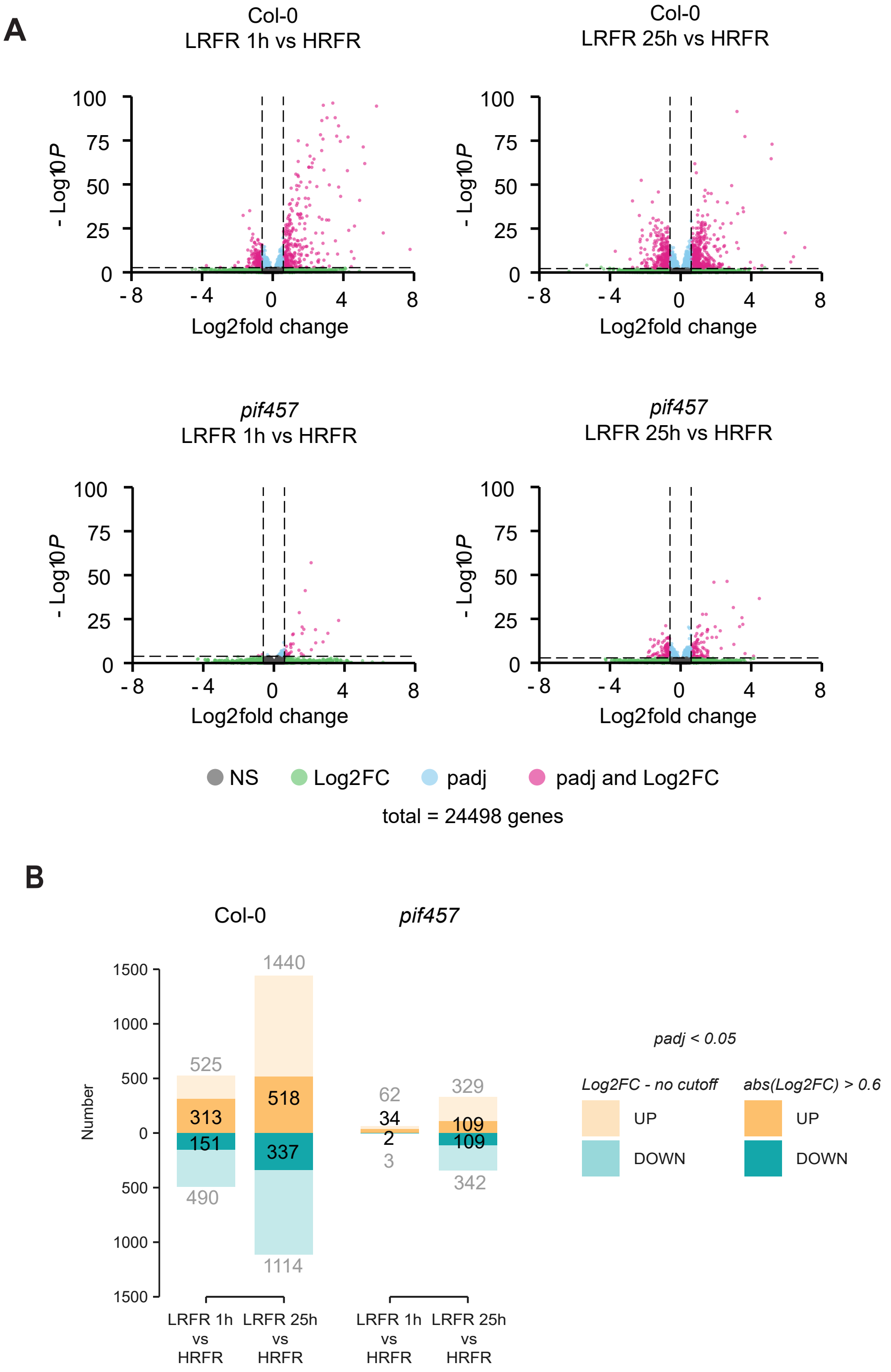

Fig. S3. PIFs promote transcriptional response to shade

A. Volcano plots of differentially expressed genes (DEGs) in comparisons of 1h and 25h of LRFR versus HRFR in Col-0 and *pif457* mutant. (padj < 0.05, abs(Log2FC) > 0.6).
B. Number of DEGs in comparisons of 1h and 25h of LRFR versus HRFR in Col-0 and *pif457* mutant at cutoff of padj < 0.05, Log2FC – no cutoff or abs(Log2FC) > 0.6.

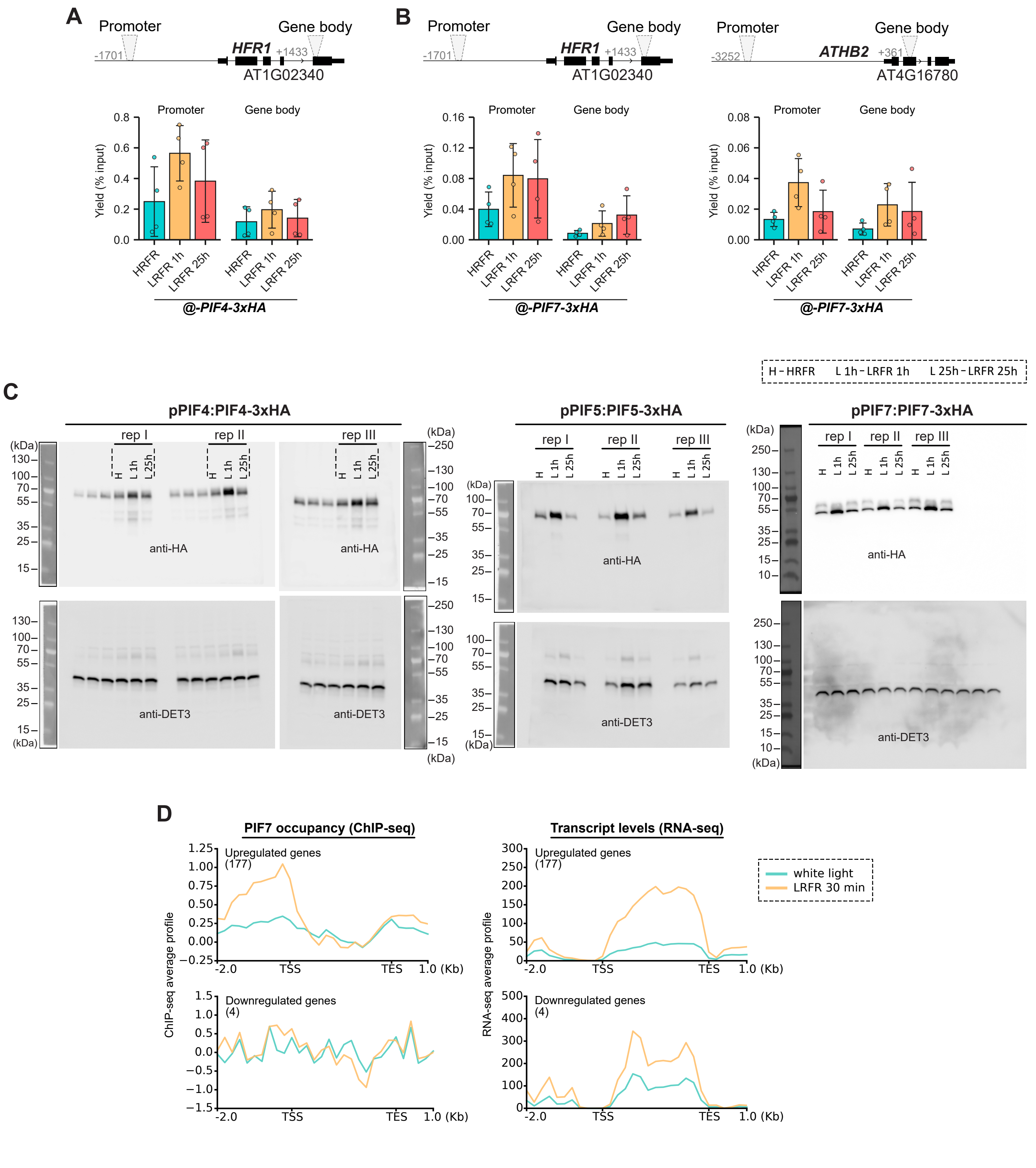

Fig. S4. Transient increase in accumulation and stability of PIFs correlates with gene occupancy in response to LRFR.

A. ChIP-qPCR of pPIF4:PIF4-3xHA line (in *pif4-101*) for *HFR1* locus.
B. ChIP-qPCR of pPIF7:PIF7-3xHA line (in *pif7-2*) for *HFR1* and *ATHB2* loci. Data are from 4 biological replicates and each biological replicate was calculated as the average of minimum two technical qPCR replicates. Bars represent the average of biological replicates. Seedlings were grown either in HRFR for 7 days (HRFR), moved to LRFR for 1h at ZT2 of day 7 (LRFR 1h) or moved to LRFR for at ZT2 of day 6 until day 7 (LRFR 25h). Samples were collected at ZT3 on day 7.
C. Western blots of pPIF4:PIF4-3xHA (in *pif4-101*, left panel), pPIF5:PIF4-3xHA (in *pif5-3*, middle panel) and pPIF7:PIF4-3xHA (in *pif7-2*, right panel) used for quantification in Fig. 3A.
D. ChIP-seq and RNA-seq average profiles of upregulated and downregulated genes as defined in Figure 1. Data is from Willige et al, 2021. Seedlings were grown in continuous white light for 6 days and then exposed to 30 min of LRFR.

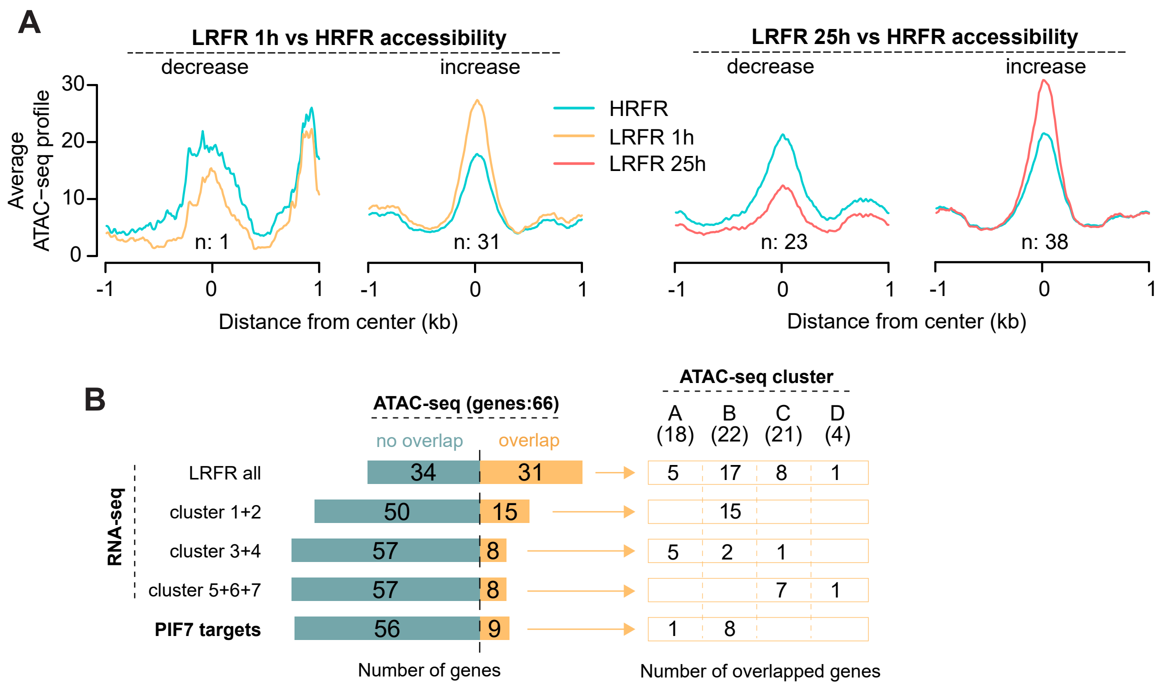

C

ATAC-seq cluster

A	B	C	D
CHIL	ACS8	ASN1	EXL3
CLC-B	BEE1	bZIP2	AT1G50650
GL22	CKX5	FLDH	AT5G01275
HFR1	CYS2	FUM2	AT5G12043
HPL1	GA20OX3	HTR11	
PDF2.1	HAT2	PLC1	
SAHH2	ATHB2	QSOX1	
TRFL1	IAA19	TPP	
TT8	PUB25	AT1G09157	
AT1G54575	SAUR23	AT1G09160	
AT1G65010	ST2A	AT1G09423	
AT2G01680	AT2G18010	AT1G26208	
AT2G05995	AT2G28060	AT1G31540	
AT2G06635	AT2G30766	AT1G74910	
AT3G50350	AT2G41760	AT2G36030	
AT4G13400	AT3G16800	AT4G16563	
AT4G19500	AT3G21330	AT4G36850	
AT5G43190	AT3G24750	AT5G15280	
	AT4G11550	AT5G44572	
	AT4G16770	AT5G52850	
	AT4G25260	AT5G59080	
	AT5G12050		

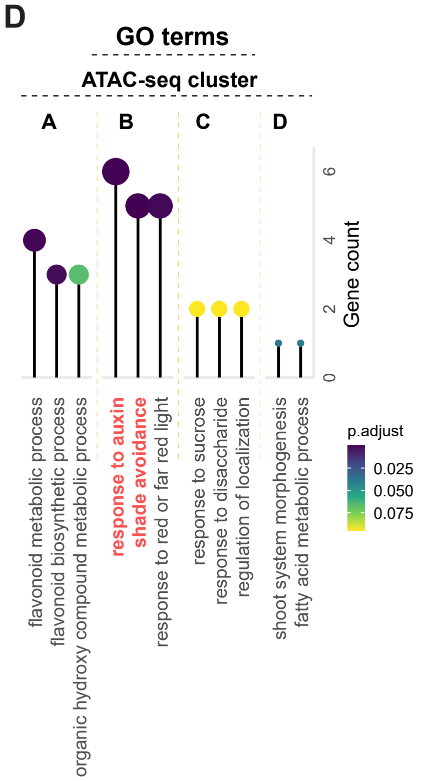

Fig. S5. Chromatin accessibility in response to LRFR affects a moderate number of genes.

A. Average ATAC-seq profiles of DARs in response to 1h of LRFR (left panel) and to 25h of LRFR (right panel). Distance from the center of the peak is expressed in kb.
B. Overlap of ATAC-seq genes, RNA-seq genes and PIF7 + shade regulated targets (left panel). Number of ATAC-seq genes per cluster that overlap with RNA-seq genes and PIF7 + shade regulated targets (right panel).
C. Table of ATAC-seq genes per cluster.
D. GO enrichment terms for ATAC-seq genes per cluster. The scale indicates gene counts. Adjusted P value for GO term is indicated by color scale.

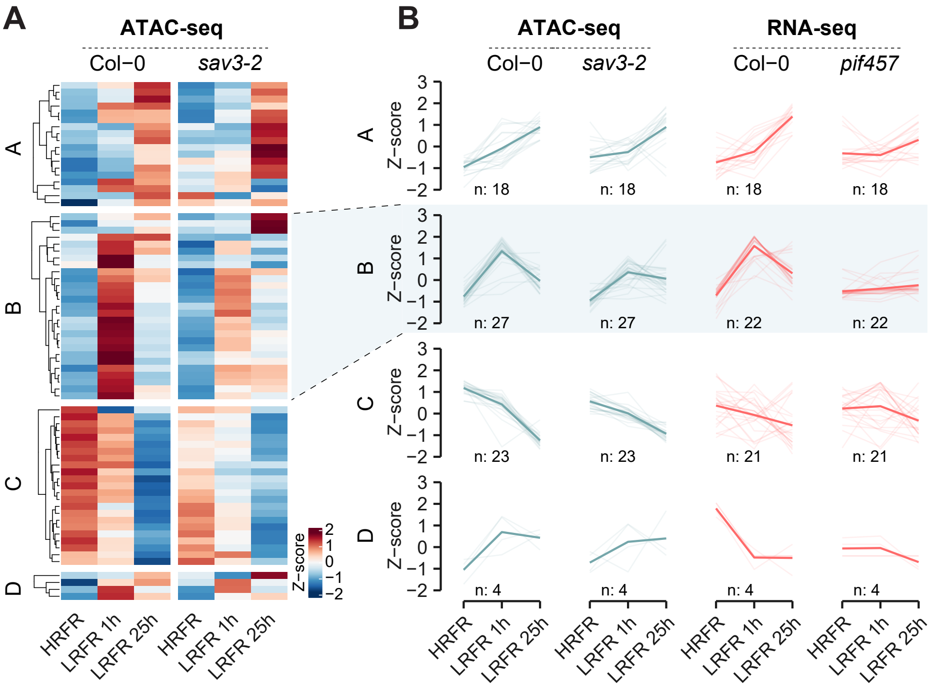

Fig. S6. Increase in chromatin accessibility in response to LRFR in *sav3-2*.

A. Heatmap of differentially accessible regions (DARs) in Col-0 and *sav3-2* in HRFR, LRFR 1h and LRFR 25h, hierarchically clustered into four distinct clusters based on ATAC-seq and RNA-seq in Col-0.
B. ATAC-seq counts of DARs in Col-0 and *sav3-2* are represented as an average z-score (in blue). The expression of genes in Col-0 and *pif457* associated with the DARs are represented as an average z-score (in red). Z-score represents row-normalized ATAC-seq chromatin accessibility (counts per million of each chromatin region are subtracted by the row mean and divided by the row standard deviation) and row-normalized RNA-seq expression (TPM of each gene is subtracted by the row mean and divided by its standard deviation). Thick line is the average trend line. Number of DARs and genes in each cluster is displayed below the line plots.

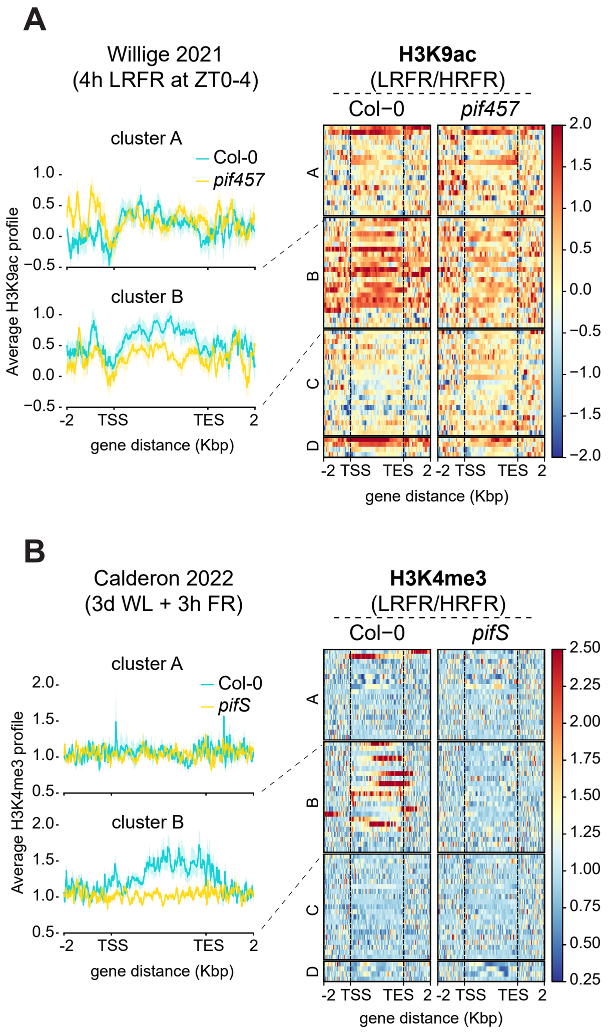

Fig. S7. Marks of active transcription increase in PIF dependent cluster under shade.

A. Average H3K9ac profile plot of cluster A and B genes as defined in Figure 4B (left panel) and H3K9ac heatmap across the gene bodies and +/- 2kbp regions (right panel). The data is from Willige et al., 2021.
B. Average H3K4me3 profile plot of cluster A and B genes as defined in Figure 4B (left panel) and H3K4me3 heatmap across the gene bodies and +/- 2kbp regions (right panel). The data is from Calderon et al., 2022.

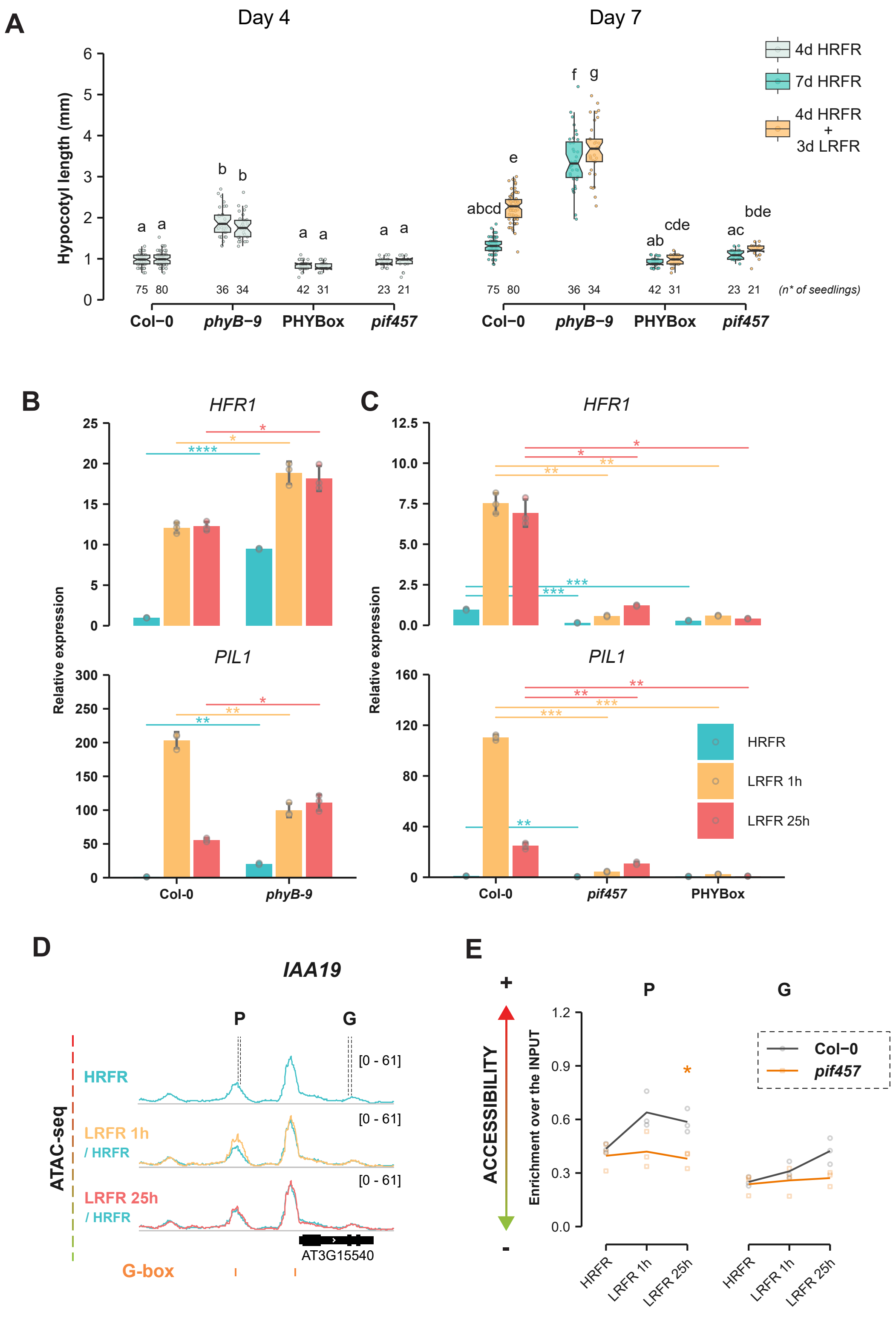

Fig. S8. Increase in chromatin accessibility of a set of shade regulated genes is induced by PIFs in response to LRFR.

A. Hypocotyl elongation of *phyB-9*, 35S:PHYB-GFP (PHYBox) line and *pif457* mutants in response to LRFR. Seedlings were grown either in HRFR for 7 days or moved to LRFR on day 4 until day 7. Hypocotyl measurements were taken on days 4 and 7. Letters represent statistical difference (ANOVA with Tukey posthoc analysis).
B. Relative gene expression of *HFR1* and *PIL1* in *phyB-9* and
C. *pif457* mutant and 35S:PHYB-GFP (PHYBox) line. Seedlings were grown either in HRFR for 7 days (HRFR), moved to LRFR for 1h at ZT2 of day 7 (LRFR 1h) or moved to LRFR at ZT2 of day 6 until day 7 (LRFR 25h). Samples were collected at ZT3 on day 7. Each bar represents the average of three biological replicates (individual replicates shown as dots) obtained by averaging three technical qPCR replicates. Error bars indicate standard deviation (SD) and asterisks represent statistical significance (Students T-test, *p<0.05, **p<0.01, ***p<0.001, ****p<0.0001).
D. IGV browser view of shade regulated gene *IAA19* with changes in chromatin accessibility in response to LRFR. ATAC-seq tracks are an average of 3 biological replicates. G-boxes are indicated in orange below the panel.
E. Chromatin accessibility of *IAA19* loci assayed by CoP-qPCR in Col-0 and *pif457* mutant. Three biological replicates with the average trend line are presented. Asterisks represent statistical significance (Students T-test, *p<0.05, **p<0.01, ***p<0.001, ****p<0.0001).

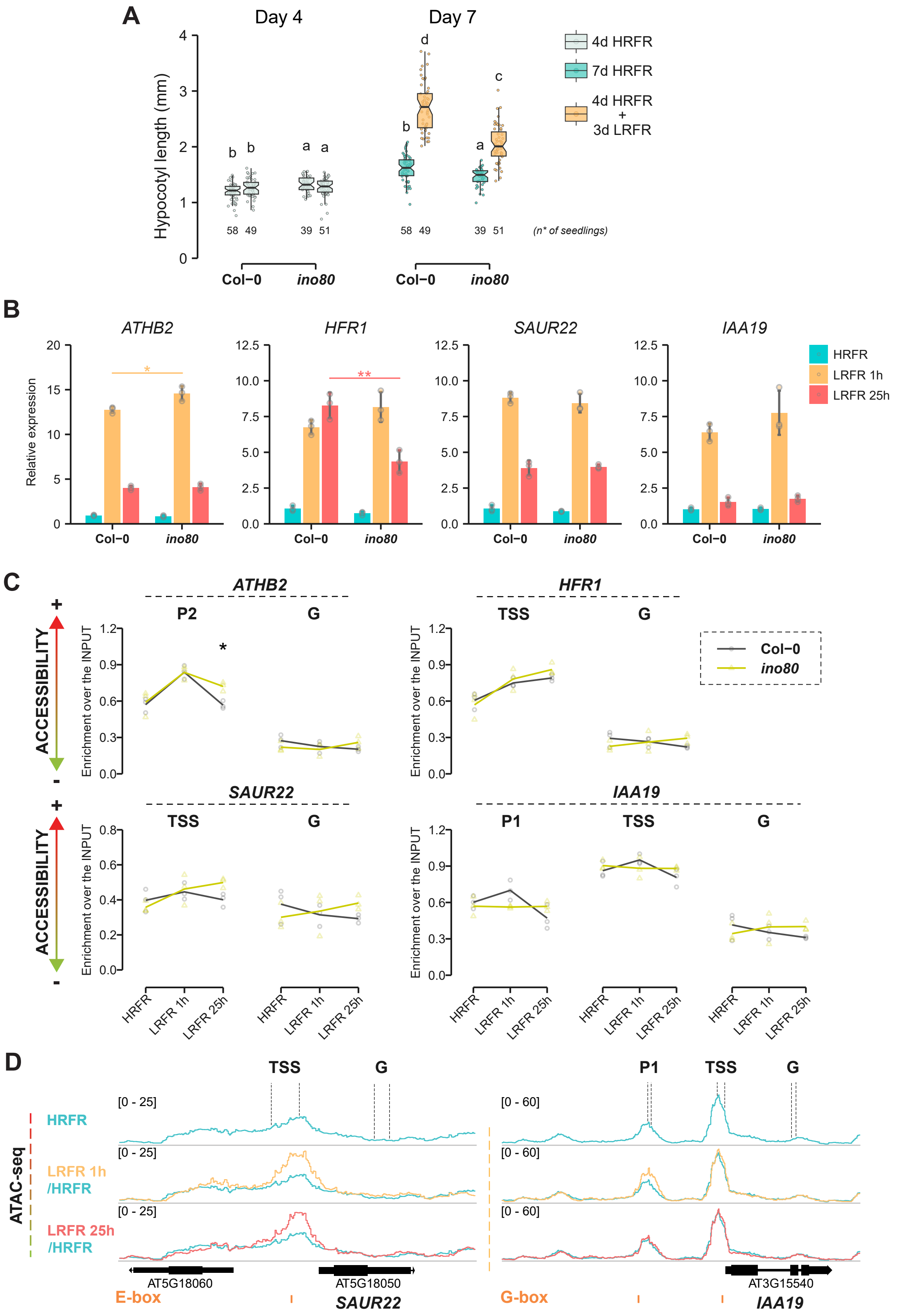

Fig. S9. Chromatin accessibility in *ino80-7* mutant

A. Hypocotyl elongation of Col-0 and *ino80-7* mutant in response to LRFR. Seedlings were grown either in HRFR for 7 days or moved to LRFR on day 4 until day 7. Hypocotyl measurements were taken on days 4 and 7. Letters denote statistical significance (ANOVA, posthoc sidak). B. Relative gene expression of *ATHB2*, *HFR1, SAUR22* and *IAA19* in *ino80-7* mutant. Seedlings were grown either in HRFR for 7 days (HRFR), moved to LRFR for 1h at ZT2 of day 7 (LRFR 1h) or moved to LRFR for at ZT2 of day 6 until day 7 (LRFR 25h). Samples were collected at ZT3 on day 7. Each bar represents the average of three biological replicates (individual replicates shown as dots) obtained by averaging three technical qPCR replicates. Error bars indicate standard deviation (SD) and asterisks represent statistical significance (Students T-test, *p<0.05, **p<0.01, ***p<0.001, ****p<0.0001). C. Chromatin accessibility of ATHB2, HFR1, SAUR22 and IAA19 loci in response to 1h and 25h of LRFR assayed by CoP-qPCR in Col-0 and *ino80-7* mutant. Three biological replicates with the average trend line are presented. Asterisks represent statistical significance (Students T-test, * p<0.05). D. IGV view of SAUR22 and IAA19 loci with changes in chromatin accessibility in response to LRFR. ATAC-seq tracks are an average of 3 biological replicates. E-boxes and G-boxes are indicated in orange below the panel.

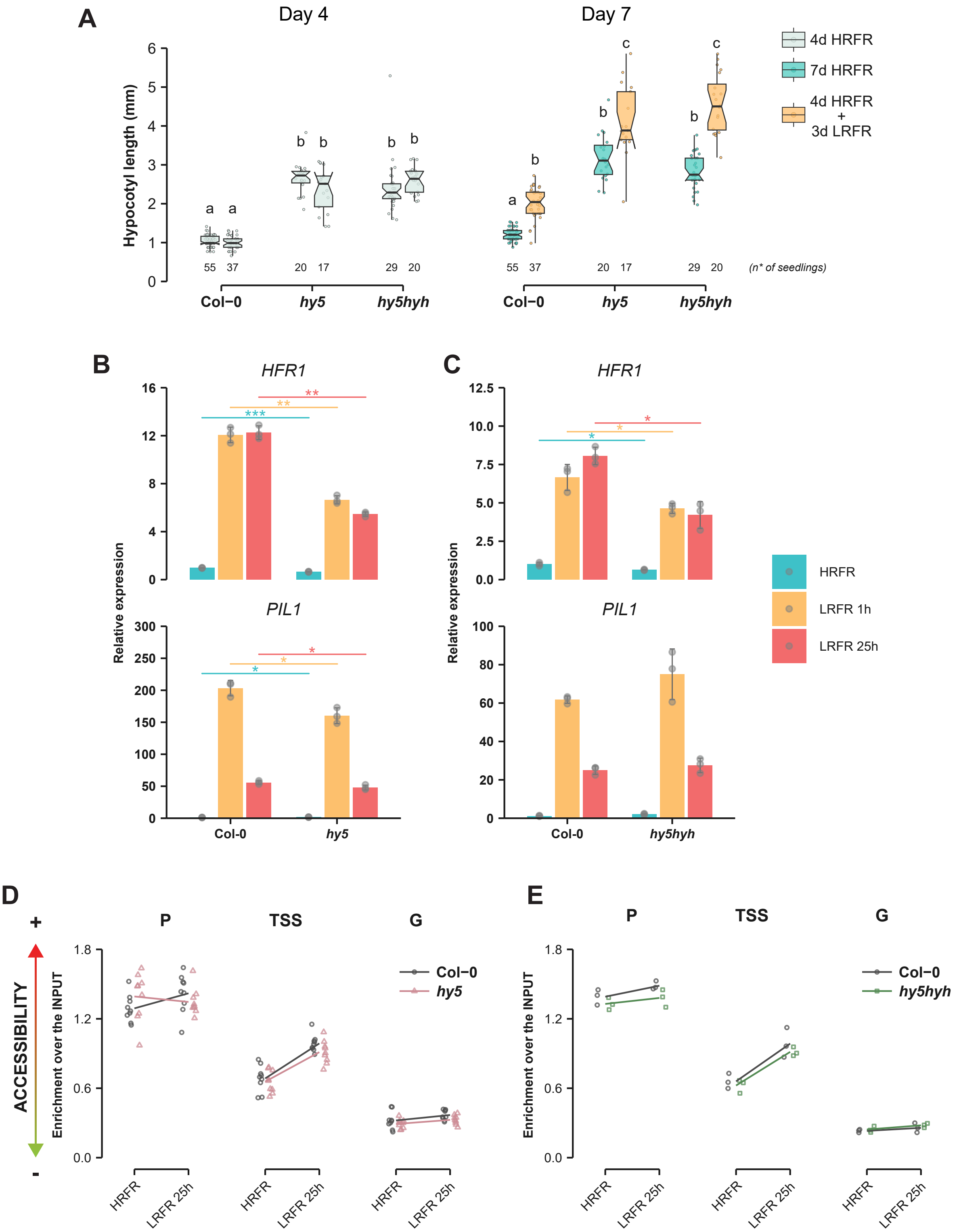

Fig. S10. Increase in chromatin accessibility of HFR1 does not depend on HY5 or HYH.

A. Hypocotyl elongation of Col-0, *hy5* and *hy5hyh* mutants in response to LRFR. Seedlings were grown either in HRFR for 7 days or moved to LRFR on day 4 until day 7. Hypocotyl measurements were taken on days 4 and 7. Letters represent statistical difference (ANOVA with Tukey posthoc analysis).
B. Relative gene expression of *HFR1* and *PIL1* in *hy5* and
C. *hy5hyh* mutants. Seedlings were grown either in HRFR for 7 days (HRFR), moved to LRFR for 1h at ZT2 of day 7 (LRFR 1h) or moved to LRFR at ZT2 of day 6 until day 7 (LRFR 25h). Samples were collected at ZT3 on day 7. Each bar represents the average of three biological replicates (individual replicates shown as dots) obtained by averaging three technical qPCR replicates. Error bars indicate standard deviation (SD) and asterisks represent statistical significance (Students T-test, *p<0.05, **p<0.01, ***p<0.001, ****p<0.0001).
D. Chromatin accessibility of HFR1 locus in response to 25h of LRFR assayed by CoP-qPCR in Col-0 and *hy5* mutant. Three independent experiments with 9 replicates and the average trend line are presented. Asterisks represent statistical significance (Students T-test, *p<0.05, **p<0.01, ***p<0.001, ****p<0.0001).
E. Chromatin accessibility of HFR1 locus in response to 25h of LRFR assayed by CoP-qPCR in Col-0 and *hy5hyh* double mutant. Three biological replicates with the average trend line are presented. Asterisks represent statistical significance (Students T-test, *p<0.05, **p<0.01, ***p<0.001, ****p<0.0001).
