# Supplementary material for: Transcriptional dynamics and chromatin accessibility in the regulation of shade-responsive genes in Arabidopsis
Source: Genome Biol. 2025 Dec 10;26:422. doi: 10.1186/s13059-025-03901-2 (PMC12690957; doi:10.1186/s13059-025-03901-2)
Supplement: Supplementary file 3 — Additional file 3: Review history [file 13059_2025_3901_MOESM3_ESM.docx]

Review History

**First round of review**

**Reviewer 1**

**Comments to author:**

Review Commons *Report 1*. https://doi.org/10.15252/rc.2025470291.

**Reviewer 2**

**Comments to author:**

Review Commons *Report 2*. https://doi.org/10.15252/rc.2025380292.

**Reviewer 3**

**Comments to author:**

Review Commons *Report 3*. https://doi.org/10.15252/rc.2025545957.

**Authors Response:**

Review Commons *Response*. https://doi.org/10.15252/rc.2025968564.

**Second round of review**

**Reviewer 1**

All my suggestions and commands have been addressed.

**Reviewer 2**

I appreciate that the authors have addressed the vast majority of my comments and am mostly satisfied that the conclusions as written accurately reflect the presented data. I have listed a few minor issues that should be addressed, but if they are addressed I believe the manuscript should be accepted for publication.

Minor comments:

It appears that an author's name (Ruben Benstein) is not spelled correctly.

Lines 35-39, "Calderon et al, 2022" is cited twice. The citation at the end of the sentence should be removed.

I appreciate that the authors reworded the phrase "key drivers" in the discussion. However, the final sentence of the introduction still proposes that PIF occupancy is "the main driving force" of transcriptional output. It may feel like splitting hairs, but there is a difference between "a major driving force" and "the major driving force" and I think the data in this manuscript support the former but not the latter, especially when taken in the context of previously published reports. I understand that the authors have highlighted differences in experimental conditions between their publications and that of Pfeiffer et al, but I still think a more accurate sentence would read something like "Our data indicate that PIF occupancy changes play a more significant role in driving gene expression changes than does remodeling of chromatin accessibility at PIF-binding sites."

A minor issue I raised in the first review has only been partially addressed. I can see that two experiments were merged in Fig. 3B but the coloring of the labels (light blue dots vs dark blue dots) is still confusing. Why are there 4 light blue dots for the LRFR 1h time point but no dark blue dots? And if there were four experiments, why are there only three dots (2 light blue and 1 dark blue) for the HRFR and LRFR 25h?

The same issue with coloring of dots is present in Fig. S4B as well.

**Reviewer 3**

The revised manuscript by Paulisic et al. provides significant improvements, including additional experimental and data analyses. Additional data and clarifications have significantly improved its quality.
The manuscript contains new experimental data to address the question of INO80 potential contribution to PIF-mediated SAR. While convincingly excluding a role of INO80 in SAR-associated chromatin acccessibility and gene expression, this addition adds to the mechanistic understanding and strengthens the study.
The study will be of significant interest for the readeship of Genome Biology. Yet, a few minor and easily-solved issues remain to be addressed.

Minor remaining issues:

* Light-mediated changes in PIF4 DNA association at target loci are shown only at two genes, used as examples. Given the general lack of reliability of ChIP-qPCR data normalization and the ambiguous representation of those performed in this study (see below), this is regrettable. Regarding PIF7, publicly available data from a related setup are used to convincingly demonstrate that PIF7 tends to be enriched at the promoter region of the set 177 PIF7-upregulated genes upon exposure to LRFR. Considering that PIF7, more than PIF4, is a major driver of SAR in Arabidopsis, this choice is acceptable.

* In Figure 3 and S4, the ChIP-qPCR results should minimally be displayed using more standard and complete representations with standard deviation from all qPCR technical and biological replicates, or showing the results of independent biological replicates separately. As it stands, the dot representation is not informative on the variations between the 3 conditions in each experiment since the corresponding dots are not connected per experiment. The representation is especially confusing for the PIL1 promoter region, not to mention the unexplained dark blue dots (not described in the legends). In addition, to ensure that ChIP signals are specific, it is necessary to probe at least one off-target, which is not the case in the data shown.

* In Figure S4D, the right panel should be labeled Transcript levels or RNA-seq, not Transcription, which cannot be estimated using RNA-seq.

* Understandably, the authors want to keep along the 4 downregulated PIF7-associated genes, as discussed, but performing a ChIP meta-profile on such a small number of genes expectedly gave no robust trend (Figure S4) and is statistically and biologically meaningless.

* The legend of Figure 4B is still incomplete; the meaning of the Z-score should be explained to aid readers.

* ChIP-seq data in Figure 1D range from negative to positive values, which is unusual. This may be due to the use of Log fold change of ratios between input and IP values. The legends should be completed.

**Authors Response**

**Point-by-point responses to the reviewers’ comments:**

Reviewer #1:
All my suggestions and commands have been addressed.

*Response: We are happy to hear that all comments have been addressed.*

Reviewer #2:

Minor comments:

It appears that an author's name (Ruben Benstein) is not spelled correctly.

*Response: Thank you for noticing, we have corrected it to Benstein.*

Lines 35-39, "Calderon et al, 2022" is cited twice. The citation at the end of the sentence should be removed.

*Response: We have removed the extra citation.*

I appreciate that the authors reworded the phrase "key drivers" in the discussion. However, the final sentence of the introduction still proposes that PIF occupancy is "the main driving force" of transcriptional output. It may feel like splitting hairs, but there is a difference between "a major driving force" and "the major driving force" and I think the data in this manuscript support the former but not the latter, especially when taken in the context of previously published reports. I understand that the authors have highlighted differences in experimental conditions between their publications and that of Pfeiffer et al, but I still think a more accurate sentence would read something like "Our data indicate that PIF occupancy changes play a more significant role in driving gene expression changes than does remodeling of chromatin accessibility at PIF-binding sites."

*Response: We have replaced the phrasing with your suggestion to more accurately reflect the findings.*

A minor issue I raised in the first review has only been partially addressed. I can see that two experiments were merged in Fig. 3B but the coloring of the labels (light blue dots vs dark blue dots) is still confusing. Why are there 4 light blue dots for the LRFR 1h time point but no dark blue dots? And if there were four experiments, why are there only three dots (2 light blue and 1 dark blue) for the HRFR and LRFR 25h?

The same issue with coloring of dots is present in Fig. S4B as well.

*Response: What appeared as darker blue dots resulted from overlapping dots. To avoid confusion and overlapping dots, both figures are now represented as bars and SD (bars show average values of biological replicates) with individual biological replicates overlayed as dots allowing all biological replicates to be clearly visible.*

Reviewer #3:

Minor remaining issues:

* Light-mediated changes in PIF4 DNA association at target loci are shown only at two genes, used as examples. Given the general lack of reliability of ChIP-qPCR data normalization and the ambiguous representation of those performed in this study (see below), this is regrettable. Regarding PIF7, publicly available data from a related setup are used to convincingly demonstrate that PIF7 tends to be enriched at the promoter region of the set 177 PIF7-upregulated genes upon exposure to LRFR. Considering that PIF7, more than PIF4, is a major driver of SAR in Arabidopsis, this choice is acceptable.

* In Figure 3 and S4, the ChIP-qPCR results should minimally be displayed using more standard and complete representations with standard deviation from all qPCR technical and biological replicates, or showing the results of independent biological replicates separately. As it stands, the dot representation is not informative on the variations between the 3 conditions in each experiment since the corresponding dots are not connected per experiment. The representation is especially confusing for the PIL1 promoter region, not to mention the unexplained dark blue dots (not described in the legends). In addition, to ensure that ChIP signals are specific, it is necessary to probe at least one off-target, which is not the case in the data shown.

*Response: This is a good suggestion, and we agree that we can clarify data representation. We decided to represent both figures in a more standard way as bars and SD (bars show average values of four biological replicates) with individual biological replicates overlayed as dots. The data of each biological replicate results from the average of technical replicates (see figure legend for details). What appeared as darker blue dots resulted from overlapping dots, this issue is fixed with the new representation of the data. While biological replicates are more variable than technical replicates, we believe that showing biological replicates is more meaningful and shows that the data is robust.
In all examples, the gene body region is a control off-target region without PIF binding sites. This is standard procedure to show binding of the transcription factor to its target sequence compared to a nearby genomic region.
To keep the style of Figure 3 consistent, we have updated the panels 3A and 3D.*

* In Figure S4D, the right panel should be labeled Transcript levels or RNA-seq, not Transcription, which cannot be estimated using RNA-seq.

*Response: Thanks for pointing out this mistake, we have now changed the title to Transcript levels (RNA-seq).*

* Understandably, the authors want to keep along the 4 downregulated PIF7-associated genes, as discussed, but performing a ChIP meta-profile on such a small number of genes expectedly gave no robust trend (Figure S4) and is statistically and biologically meaningless.

* The legend of Figure 4B is still incomplete; the meaning of the Z-score should be explained to aid readers.
 *Response: We have updated all the legends with the meaning of Z-score for both ATAC-seq and RNA-seq.*

* ChIP-seq data in Figure 1D range from negative to positive values, which is unusual. This may be due to the use of Log fold change of ratios between input and IP values. The legends should be completed.

*Response: We have updated the legends of Figure 1D to reflect that ChIP was normalized to Col-0 IgG using bamCompare resulting in positive and negative values. The data used for plotting is from Willige et al., 2021 deposited here: GSE139296.
In addition, the legend is updated with details of statistical test used for Figure 1B with minor correction of significance for comparison between Col-0 and pif457 in HRFR condition.*
